# Differential Expression of CHL1 Gene during Development of Major Human Cancers

**DOI:** 10.1371/journal.pone.0015612

**Published:** 2011-03-07

**Authors:** Vera N. Senchenko, George S. Krasnov, Alexey A. Dmitriev, Anna V. Kudryavtseva, Ekaterina A. Anedchenko, Eleonora A. Braga, Irina V. Pronina, Tatiana T. Kondratieva, Sergey V. Ivanov, Eugene R. Zabarovsky, Michael I. Lerman

**Affiliations:** 1 Laboratory of Structural and Functional Genomics, Engelhardt Institute of Molecular Biology, Russian Academy of Sciences, Moscow, Russia; 2 Laboratory of Molecular Diagnosis, Russian State Genetics Center GosNIIgenetika, Moscow, Russia; 3 Blokhin Cancer Research Center, Russian Academy of Medical Sciences, Moscow, Russia; 4 Cardiothoracic Surgery Department, NYU Langone Medical Center, New York, New York, United States of America; 5 Department of Microbiology, Tumor and Cell Biology, Department of Clinical Science and Education, Sodersjukhuset, Karolinska Institute, Stockholm, Sweden; 6 Hematology Branch, National Heart, Lung, and Blood Institute, National Institutes of Health, Bethesda, Maryland, United States of America; Baylor College of Medicine, United States of America

## Abstract

**Background:**

*CHL1* gene (also known as *CALL*) on 3p26.3 encodes a one-pass trans-membrane cell adhesion molecule (CAM). Previously CAMs of this type, including L1, were shown to be involved in cancer growth and metastasis.

**Methodology/Principal Findings:**

We used Clontech Cancer Profiling Arrays (19 different types of cancers, 395 samples) to analyze expression of the *CHL1* gene. The results were further validated by RT-qPCR for breast, renal and lung cancer. Cancer Profiling Arrays revealed differential expression of the gene: down-regulation/silencing in a majority of primary tumors and up-regulation associated with invasive/metastatic growth. Frequent down-regulation (>40% of cases) was detected in 11 types of cancer (breast, kidney, rectum, colon, thyroid, stomach, skin, small intestine, bladder, vulva and pancreatic cancer) and frequent up-regulation (>40% of cases) – in 5 types (lung, ovary, uterus, liver and trachea) of cancer. Using real-time quantitative PCR (RT-qPCR) we found that *CHL1* expression was decreased in 61% of breast, 60% of lung, 87% of clear cell and 89% papillary renal cancer specimens (*P*<0.03 for all the cases). There was a higher frequency of *CHL1* mRNA decrease in lung squamous cell carcinoma compared to adenocarcinoma (81% vs. 38%, *P* = 0.02) without association with tumor progression.

**Conclusions/Significance:**

Our results suggested that *CHL1* is involved in the development of different human cancers. Initially, during the primary tumor growth *CHL1* could act as a putative tumor suppressor and is silenced to facilitate *in situ* tumor growth for 11 cancer types. We also suggested that re-expression of the gene on the edge of tumor mass might promote local invasive growth and enable further metastatic spread in ovary, colon and breast cancer. Our data also supported the role of *CHL1* as a potentially novel specific biomarker in the early pathogenesis of two major histological types of renal cancer.

## Introduction

Cancer-associated genes fall into two main categories: cancer-causing genes that drive malignant transformation and maintain tumor growth, and cancer progression genes that orchestrate local invasion and further spread of metastatic cells and growth of distant metastases [1], [2], [3]. The *CHL1* gene – close homolog of L1, also known as *CALL* - cell adhesion L1-like (GenBank Accession No. NM_006614.2) encodes a one-pass trans-membrane cell adhesion molecule (CAM) capable of both homotypic and heterotypic binding. The protein encoded by this gene is a member of the L1 gene family of neural cell adhesion molecules. It is a neural recognition molecule that may be involved in signal transduction pathways. *CHL1* is expressed in normal tissues besides the brain and is expressed in a variety of human cancer cell lines and primary tumor tissues [4], [5]. It was also shown that the gene is involved in general cognitive activities (g/IQ) [6], [7] and some neurological diseases (i.e. schizophrenia [8]). The deletion of one copy of this gene may be responsible for mental defects in patients with 3p- syndrome. Recently several CAMs including L1 were shown to be involved in cancer growth and metastasis [9], [10]. *CHL1* is located at 3p26, a region that is shown to harbor a candidate for prostate cancer susceptibility in Finnish prostate cancer families, although no mutations were detected in the coding part of the gene [11]. Thus, these reports suggest that *CHL1* plays a role in cancer development [12], not only in neuronal activities. Previously, in collaboration with Dr. Helen S. Smith, we performed a deletion mapping of the short arm of chromosome 3 on a panel of breast cancers and delineated three regions as harboring breast cancer candidate tumor suppressor genes (TSG), namely, 3p24-26, 3p21-22, and 3p12-13 [13], [14], [15], [16]. Then we cloned the *CHL1 (CALL)* gene in 1997/1998 and analyzed its expression in mouse development and performed extensive bioinformatics analysis [5].

Here we provided a comprehensive study of *CHL1* mRNA expression using two methods. Qualitative analysis was performed using Clontech Cancer Profiling Arrays, and further real time quantitative PCR (RT-qPCR) was employed for validation of the microarray data for three major cancer types: non-small cell lung cancer (NSCLC), breast cancer (BC), and renal cell carcinomas (RCC). Our results suggested a dual role of the *CHL1* in tumorigenesis: it may contribute to initial tumor growth and then to progression and finally tumor spread/metastasis. The data further supported the role of *CHL1* as a potentially novel specific biomarker in the early pathogenesis of two major histological types of renal cancer.

The work is dedicated to the memory of Dr. Helen S. Smith.

## Results

### *In silico* analysis of CHL1 expression in normal and tumor tissues

The vast public expression databases allow to detect and quantify the expression of most if not all known RefSeq genes (∼20,000) in normal and tumor tissues. We used several public web-based servers to analyze mouse and human *CHL1* expression [17], [18], [19], [20]. The data shows that *CHL1* is expressed in many normal adult and fetal tissues besides the brain and peripheral nervous system [17], [19]. Variable expression was seen in many tumors; it was especially high in a melanoma cell line G361. According to Oncomine [18] preliminary data based on microarray analysis, the *CHL1* expression also varies in several major cancer types – renal [21], [22], cervical [23], colon [24], [25], ovary [26], lung [27], [28], stomach [29] and breast [30], [31] cancer. The Oncomine also showed co-expression of *CHL1* with another known cancer metastasis-associated gene, lysyl oxidase (*LOX*) [32] in metastatic melanoma.

### Investigation of CHL1 expression with Cancer Profiling Arrays

**Figure 1:**
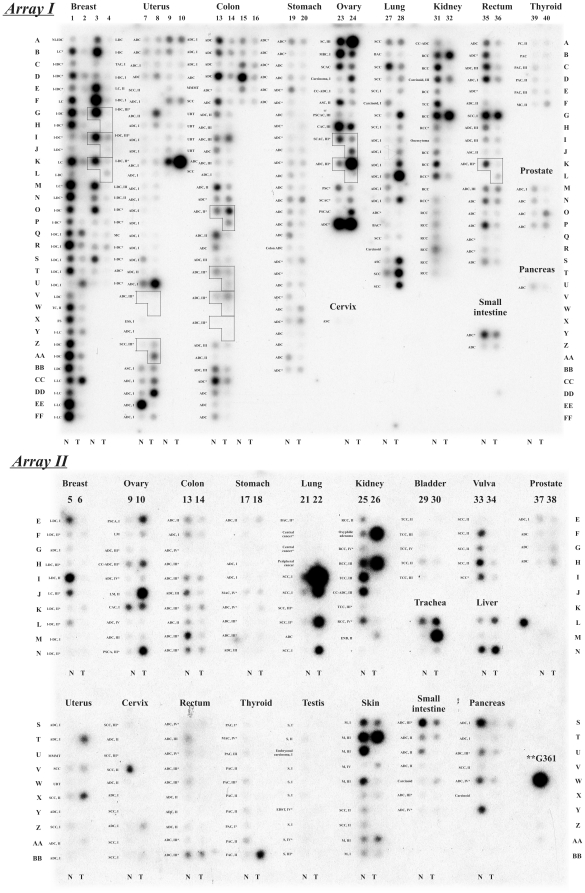
Results of microarray analysis of *CHL1* mRNA level in different types of cancer. Abbreviations used: ADC - adenocarcinoma, ASC - adenosquamous carcinoma, BAC - bronchiolo-alveolar adenocarcinoma, C - carcinoma, CAC - cystadenocarcinoma, CC-ADC - clear cell adenocarcinoma, EDST - endodermal sinus tumor, ENB - epithelial nephroblastoma, ESS - endometrial stromal sarcoma, FAC - follicular adenocarcinoma, FS - fibrosarcoma, I-DC - infiltrating ductal carcinoma, I-IDC - infiltrating intraductal carcinoma, I-LC - infiltrating lobular carcinoma, LC - lobular carcinoma , LDC - mixed lobular-ductal carcinoma, LM - leiomyoma, M - malignant melanoma, MAC - mucinous adenocarcinoma, MBC - mucinous borderline carcinoma, MC - medullary carcinoma, MMMT - malignant mixed Mullerian tumor, NI-IDC - noninfiltrating intraductal carcinoma, PAC - papillary adenocarcinoma, PC - papillary carcinoma, PSC - papillary serous carcinoma, PSCA - papillary serous cystadenoma, PSCAC - papillary serous cystadenocarcinoma, RCC - renal cell carcinoma, S - seminoma, SC - serous carcinoma, SCAC - serous cystadenocarcinoma, SCC - squamous cell carcinoma, TAC - tubular adenocarcinoma, TC - tubular carcinoma, TCC - transitional cell carcinoma, UBT - uterus benign tumor. Asterisks (*) show samples with metastases. **G361 – a melanoma cell line. The boxed samples indicate a matched normal (left) - primary tumor (bottom right) pair with an associated metastatic sample (upper right corner of a box). T - tumor samples; N - matched normal control specimens.

We used Cancer Profiling Arrays I and II (Clontech) to test the *CHL1* expression in a large sample of human primary tumors including breast, lung, kidney, ovary, colon, stomach and others (Fig. 1). Only 395 samples of 486, including 90 metastatic tumors and 12 metastases were informative. We first showed that the change of *CHL1* expression in all studied tumors compared to the matched non-cancerous (normal) tissues was statistically significant (*P*<0.05, Fisher exact test or χ^2^ criteria).

#### Down-regulation

**Figure 2:**
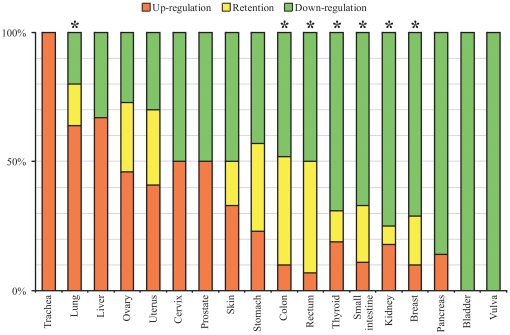
Distribution of primary tumors with different *CHL1* mRNA level alterations. Fraction of tumors with *CHL1* up-regulation is showed with red, down-regulation – green, mRNA level retention – yellow. Data revealed with Clontech Microarray analysis. Asterisks (*) show statistically significant differences between frequencies of *CHL1* expression changes with up- and down-regulation.

As demonstrated by Cancer Profiling Arrays data in Figures 1 and 2, a high percentage of patients displayed a down-regulation of *CHL1* expression in breast, kidney, rectum, colon, thyroid, stomach, skin, small intestine, bladder, vulva and pancreatic cancer. The results of the microarray data analysis were presented for 11 types of cancer in Table 1. In total, a statistically significant decrease of *CHL1* expression was found in breast cancer - 71% (45 of 63 cases), colon - 48% (23 of 48), rectum - 50% (14 of 28), thyroid - 69% (11 of 16), kidney - 75% (21 of 28) and small intestine – 67% (6 of 9) cancers. Importantly, a statistically significant increase of down-regulation frequency was shown in samples with metastases compared to samples without metastases in colon (83% vs. 36%, *P* = 0.01) and rectum (75% vs. 31%, *P* = 0.05) cancers. The same tendency was found in ovary cancer (60% vs. 19%, *P* = 0.17).

**Table 1:** Summary of *CHL1* expression in 11 tumor types as measured with the Clontech Cancer Profiling Arrays I and II.

Cancer	No metastases		With metastases		* *P*	Totally		** *P*
	Down	Up	Down	Up		Down	Up	
*Breast*	**64** (25/39)	13 (5/39)	**83** (20/24)	4 (1/24)	0.25	**71** (45/63)	10 (6/63)	**<0.01**, χ^2^
*Uterus*	**29** (12/42)	**41** (17/42)	50 (1/2)	50 (1/2)	0.62	**30** (13/44)	**41** (18/44)	0.37
*Ovary*	**19** (4/21)	**52** (11/21)	**60** (3/5)	20 (1/5)	*0.17*	**27** (7/26)	**46** (12/26)	0.25
*Colon*	**36** (13/36)	11 (4/36)	**83** (10/12)	8 (1/12)	**0.01**	**48** (23/48)	10 (5/48)	**<0.01**
*Rectum*	**31** (5/16)	6 (1/16)	**75** (9/12)	8 (1/12)	**0.05**	**50** (14/28)	7 (2/28)	**<0.01**
*Stomach*	**28** (5/18)	17 (3/18)	**59** (10/17)	**29** (5/17)	**0.02**	**43** (15/35)	23 (8/35)	0.13
*Thyroid*	**58** (7/12)	19 (3/12)	**100** (4/4)	0 (0/4)	0.30	**69** (11/16)	19 (3/16)	**0.01**
*Lung*	18 (4/22)	**64** (14/22)	33 (1/3)	67 (2/3)	0.65	20 (5/25)	**64** (16/25)	**<0.01**
*Kidney*	**70** (16/23)	18 (5/23)	**100** (5/5)	0 (0/5)	0.36	**75** (21/28)	18 (5/28)	**<0.01**
*Trachea*	0 (0/1)	100 (1/1)	0 (0/2)	100 (2/2)	×	0 (0/3)	100 (3/3)	×
*Small intestine*	**60** (3/5)	20 (1/5)	**75** (3/4)	0 (0/4)	0.64	**67** (6/9)	11 (1/9)	**0.05**

Note: *P – p-parameter, characterized the difference between group with metastases and group without metastases, taking into account 3 possible events: decrease, increase and retention of mRNA level in tumor samples comparing to adjusted normal samples. P-parameter calculated using χ^2^ method.

**P – p-parameter, characterized the difference between group of tumor samples and group of adjusted normal samples, taking into account prevalent process (decrease or increase of mRNA level). P-parameter calculated using Fisher exact test for all cancers, except breast cancer.

Bold – the most significant changes.

#### Up-regulation

The *CHL1* up-regulation (frequency from 20% to 100%) was found in lung, ovary, uterus, liver, skin, prostate, stomach, cervix and trachea cancers. However, the increase of the *CHL1* mRNA level was statistically significant only in lung cancer −64% (16 of 25, *P*<0.01). The majority of such cases (14 of 22, P<0.01) were found in different histotypes of NSCLC (ADC, BAC, SCC) at the Stage I. Also we observed several cases of *CHL1* up-regulation in metastatic tumors (stomach, lung, trachea, ovary and uterus, Table 1). Thus, the cases with *CHL1* up-regulation could serve as examples of *CHL1* involvement both in initial and possibly in further progression and invasive tumor growth.

#### Deregulation

In uterus and ovary cancer the frequency of up- and down-regulation was close (41% and 30%, 46% and 27%, respectively). In ovary cancer the down-regulation was a prevalent event (52%) in samples without metastases, on the contrary the up-regulation was prevalent (60%) in the group of metastatic tumors. In stomach cancer a statistically significant change of *CHL1* expression (both up- and down-regulation) was shown in the group with metastases relative to the group without metastases (88% vs. 45%, *P* = 0.02).

#### Metastases

We observed re-expression of the *CHL1* in 4 of 12 metastases (first coordinate) along with low *CHL1* mRNA level in primary tumor (second coordinate): in ovary (24K/24L), colon (14O/14P, 14V/14W) and breast (4I/4J, Fig. 1, Array I). In addition, we also found silencing of the gene expression in both metastasis and primary tumors, for example, breast cancer (4G/4H, 4K/4L).

### The CHL1 expression in breast, lung and renal cancer tissues studied using RT-qPCR

**Figure 3:**
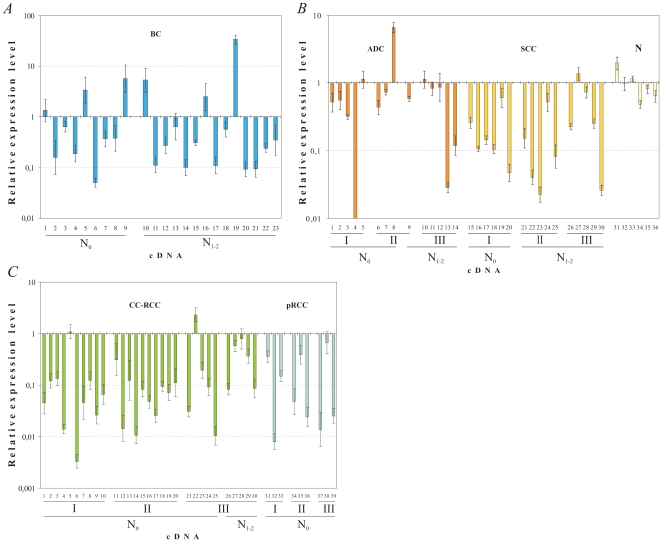
Expression profiling of *CHL1* in primary breast, lung and renal tumors. **A.** The relative *CHL1* mRNA level (R) in breast cancer (BC). N_0_ – without metastases, N_1–2_ – metastases in regional lymph nodes. Samples #1, 2 (Stage I), #3-22 (Stage II), #23 (Stage IV); samples #3-9 (Grade 1), #10-21 (Grade 2). **B.** The relative *CHL1* mRNA level (R) in lung cancer (NSCLC). SCC – lung squamous cell carcinomas, ADC – lung adenocarcinomas, N – normal samples from cancer free healthy donors; N_0_ – without metastases, N_1–2_ – metastases in regional lymph nodes; I, II and III – Stages. **C.** The relative *CHL1* mRNA level (R) in renal cancer (RCC). CC-RCC – clear cell renal carcinomas, pRCC – papillary renal carcinomas; N_0_ – without metastases, N_1–2_ – metastases in regional lymph nodes; I, II and III – Stages.

The *CHL1* mRNA content was decreased in the majority of studied tumor samples compared to normal samples but in some tumor samples the *CHL1* expression was up-regulated (Fig. 3. A, B and C).

#### Breast cancer (BC)

We found that the *CHL1* mRNA level was decreased in 61% (14 of 23, *P*<0.03), increased in 22% (5 of 23) and not changed in 17% (4 of 23) of samples. Maximal decrease of the *CHL1* mRNA level was 20-fold, maximal increase was 34-fold. There was no evident correlation between the change of the *CHL1* expression and the tumor progression (Fig. 3 A).

#### Non-small cell lung cancer (NSCLC)

The *CHL1* mRNA level was decreased in 60% (18 of 30, P<0.02) and was normal in 33% (10 of 30), i.e. less than 2-fold changes. The decrease or increase of the mRNA level was detected neither in lung non-cancerous (normal) matched tissues nor in tissues from cancer free healthy donors. However, for two histological subtypes of NSCLC (ADC and SCC) the frequency of the mRNA changes was different. Down-regulation was observed in 38% (5 of 14) of ADC samples. The increase of the *CHL1* mRNA (7-fold) was detected only in one ADC sample. On the contrary, in SCC samples the *CHL1* expression was significantly decreased in 81%, (13 of 16, *P*<0.02). *LD* (level of mRNA decrease) varied from 2 to 100-fold in ADC and from 2 to 44-fold in SCC. There was a more significant increase of *FD* (frequency of mRNA decreases) values in SCC as compared to ADC (81% vs. 38%, *P* = 0.02) without noticeable association with tumor progression (Fig. 3, B and Table 2).

**Table 2:** The frequency (*FD*) and the level (*LD_av_*) of the *CHL1* mRNA decrease in lung cancer (ADC and SCC types).

Group		ADC	SCC
Without metastases	*FD*,%	38 (3/8)	83 (5/6)
	a *LD_av_*	9 (2—100)	9 (4—21)
	b *P*	nsv	<0.03
With metastases	*FD*,%	33 (2/6)	80 (8/10)
	*LD_av_*	17 (2—35)	10 (2—44)
	*P*	nsv	<0.02
Total	*FD*,%	38 (5/14)	81 (13/16)
	*LD_av_*	12 (2—100)	10 (2—44)
	*P*	nsv	<0.02

a The *LD_av_* values are shown in fold-change ratios.

b *P* reflects the difference between group of tumor samples and group of adjusted normal samples.

The *LD* range is given in parentheses. nsv - not statistically valid.

#### Clear cell renal cell carcinoma (CC-RCC), papillary renal cell carcinoma (pRCC) and renal carcinoma cell lines

A significant decrease (from 3 to 302-fold) of *CHL1* mRNA was detected in 87% (26 of 30, *P*<0.01) of CC-RCC specimens and 89% (8 of 9, *P*<0.02) of pRCC specimens with *LD_av_* (geometric mean of LD) equal to 18 and 19-fold respectively (Fig. 3, C). Therefore we could conclude that the frequency and the average level of the *CHL1* expression decrease were similar for two major histological types of renal cancer, CC-RCC and pRCC. The *LD_av_* value was significant in all RCC tumors at all developmental stages independent of metastasis presence (Table 3). In CC-RCC with or without metastases, the *FD* and *LD_av_* values were similar.

**Table 3:** The frequency (*FD*) and the level (*LD_av_*) of the *CHL1* mRNA decrease in renal cancer (CC-RCC).

CC-RCC	Stage			Total
	I	II	III	
*FD*,%	90 (9/10)	100 (10/10)	70 (7/10)	87 (26/30)
*LD_av_*	24 (8—302)	17 (3—93)	13 (3—96)	18 (3—302)
*P*	<0.02	<0.02	<0.02	<0.01

**Figure 4:**
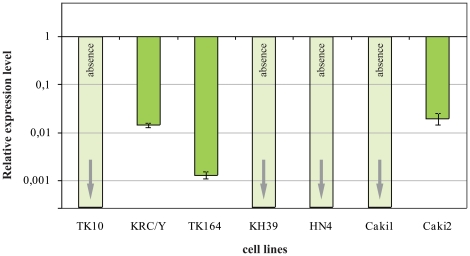
The relative expression levels of *CHL1* in CC-RCC cell lines. The mRNA level of the target gene was normalized to the reference genes *RPN1* and *GUSB*.

The estimates of the *CHL1* mRNA levels in seven renal cancer cell lines revealed strong down-regulation of this gene: 80-fold (Caki2, KRC/Y), about 1000-fold (TK164) and total silencing (TK10, KH39, HN4, Caki1, Fig. 4).

### Comparison of microarrays and RT-qPCR data for breast, renal and lung cancer

The microarray data for 61 BC, 23 RCC and 25 NSCLC samples were compared with RT-qPCR data for 23 BC, 30 CC-RCC and 30 NSCLC samples. In our array study a significant *CHL1* down-regulation was shown for most of RCC samples; up-regulation was observed in 3 cases only. Our results also showed the down-regulation of *CHL1* in most of BC samples independent of the presence of metastases and up-regulation in 7 tumors only. Almost the same results were obtained using RT-qPCR. There were similarities between array and quantitative data for renal and breast cancer (Table 4).

**Table 4:** The *CHL1* mRNA changes using microarrays and RT-qPCR.

Cancer	Changes	Frequency of *CHL1* mRNA changes, %	
		Microarrays	qPCR
BC	Increase	11 (7/61)	22 (5/23)
	Decrease	75 (46/61)	61 (14/23)
RCC	Increase	13 (3/23)	3 (1/30)
	Decrease	78 (18/23)	87 (26/30)

Cancer Profiling Arrays I and II include a very heterogeneous group of lung cancer with different histological subtypes: BAC, ADC, SCC, carcinoid with Stage I and II, only two metastatic tumors and limited number of specimens of each subtype. Overall, there were 15 SCC and 5 ADC which we could compare with RT-qPCR data. We found an up-regulation in 11 SCC (6 of 11 were identified as Stage I) and 4 ADC (3 ADC were Stage I as well); down-regulation in 2 SCC (13%) and 1 ADC (25%, Fig. 1). Recently, we showed an up-regulation of several TSG on 3p in lung ADC at Stage I. These tumors were characterized with high differentiation grade [33]. On the other hand, according to the RT-qPCR data frequency of decreased expression was 38% (5 of 14 cases) in ADC and 81% (13 of 16 cases, P<0.02) in SCC (see Table 2). An up-regulation was detected only in 7% (1 of 14 cases) ADC and never in SCC.

## Discussion

*CHL1*, located at 3p26.1, belongs to the family of cell adhesion molecules (CAM) – cell surface proteins mediating cell-cell and cell-matrix interactions. Alterations in CAM expression (including *CHL1*) and functions have been implicated in development of different tumor types, for example, melanoma [34], ovary [9], [35], prostate [11] and colon cancer [36]. According to [9], the evaluation of LOH patterns in serous epithelial ovarian cancer (EOC) suggested that *CHL1* is a tumor suppressor candidate (TSG). The studies published by us and other authors (see Introduction) suggested that the *CHL1* gene could be one of the putative tumor suppressor genes localized on human chromosome 3 [12]. However, overexpression of *CHL1* was observed in serous EOC samples [9]. Furthermore, L1 CAM overexpression in malignant melanoma was shown to be associated with metastases [34].

According to Oncomine preliminary microarray expression data [17] along with the prevalent *CHL1* down-regulation in several tumors (RCC, lung SCC, colon ADC), the overexpression of CHL1 was found in melanoma. The differential expression was observed in lung ADC [26], cervical [22] and breast [29], [30] cancer.

Based on this data, we hypothesized that CHL1 and other recognition receptors of this type might have dual roles in cancer: in early pre-invasive growth they could serve as TSG and are silenced; later at invasion and metastasis stages these genes might be re-expressed on the edge of the tumor to drive local invasion and enable metastatic spread.

This hypothesis was analyzed in the current study using a combination of preliminary expression screening in 19 different types of epithelial tumors with commercial microarrays (altogether 395 informative samples, Table 1) and evaluation of the *CHL1* mRNA expression in primary tumors using RT-qPCR. This method is widely used to corroborate disease-associated expression signatures derived from microarrays. Furthermore, this technology is well suited for translating microarrays data into accurate and quantitative, clinically useful assays [37].

We showed here that the expression of *CHL1* was deregulated in major epithelial malignancies (76%, *P*<0.01, including 54% down-regulation cases according to microarray data). Statistically significant *FD* values were shown for breast, colon, rectum, thyroid, kidney and small intestine cancer (Table 1). For three social significant/important cancer types – breast, kidney and lung microarray data were validated by RT-qPCR. There was a good concordance between data of two methods for kidney and breast cancer. According to Oncomine microarray data the significant decrease of *CHL1* expression level in CC-RCC samples was shown as well.

Clontech Microarray (overexpression in 64% lung cancer samples) and RT-qPCR (down-regulation in 38% of ADC and in 81% of SCC samples) were not in concord because different cancer subtypes were present in studied microarray samplings. The disagreement between arrays and RT-qPCR data for NSCLC could also result from non-homogeneous samples with different content of normal cells as well as limited number of specimens and may be statistically non-significant. Although these data are not statistically valid they could reflect important trends and associations.

However there was a rather good agreement between our quantitative results for lung cancer and Oncomine data [17] for two major lung cancer histotypes – ADC and SCC.

It is important to note that Oncomine exploits microarrays based on completely different platform than Clontech Cancer Profiling Arrays. Traditional microarrays (Affymetrix, Agilent) contain a number of various gene probes immobilized on glass slides. Only one cDNA sample can be hybridized with the slide. On the contrary, Clontech Cancer Profiling Arrays contain a number of immobilized cDNA samples from various tumor and normal tissues. Oncomine includes traditional microarrays data making possible genome-wide analysis of a limited number of samples and Cancer Profiling Arrays enable analysis of one gene in many tumors in one experiment.

According to the Clontech Microarray data, the mRNA level increase was observed for several tumor types – uterus, ovary, colon, stomach, thyroid, lung, kidney, and trachea – mainly for non-metastatic tumors. However, also there were frequent cases of the *CHL1* mRNA level increase in metastatic tumors, for example, in stomach and lung cancer.

Moreover in four metastases (4I, 14O, 14V, 24K) of 12 available for analysis cases (i.e. when a primary tumor and metastasis for the same patient were accessible) we detected an increased *CHL1* expression in metastasis compared to primary tumor (ovary, colon and breast). Similar results were recently reported for the metastasis-associated gene lysyl oxidase (*LOX*), whose expression was associated both with tumor suppression and tumor progression depending on transformation status [32]. The overexpression of another cell adhesion molecule L1 gene was associated with metastasis in malignant melanoma [34].

Cancer is a fatal disease whereby invasive local tumor growth and metastatic spread to distant vital organs resulting in dormant and/or active growth and inevitable death of patients. Contrary to previous models new evidence suggested that metastatic cells might be created already during initial growth of a primary local tumor. These cells then succeed in cell migration/invasion, embolization, survival in the circulation, arrest in a distant capillary bed, and extravasation into and multiplication within the distant organ parenchyma. Failure at any of these steps could block the entire metastatic process and may lead to “dormant cancer cells and dormant micrometastases”. Surgical removal of the primary tumor might then lead to active growth [38]. Because tumor spread is responsible for the majority of deaths of cancer patients, the development of therapeutic agents that inhibit tumor metastasis is of paramount importance [39], [40], [41], [42], [43], [44], [45], [46], [47].

One of us predicted previously [5] that the cytoplasmic end of CHL1 protein might interact with the cytoskeleton and might induce/regulate filopodia formation driving tumor cell migration and invasion [41], [45], [46]. *CHL1* behavior in cancer is thus strikingly similar to *L1* [10], [40] and *LOX* which both work through the actin network.

This study suggested that *CHL1* might contribute to cancer invasive growth and metastasis. It might act either as a tumor suppressor (early growth) or oncogene (invasive and metastatic growth, Fig. 1, Table 1). *CHL1* therefore could belong to the new rapidly growing category of cancer genes that may function either as TSGs or oncogenes [32], [41], [43], [46], [47], [48]. During initial growth *CHL1* is not expressed (silenced) in tumor cells to facilitate *in situ* tumor growth. Re-expression of *CHL1* on the edge of the tumor mass and around tumor vessels could promote migration and local invasive growth and furthermore allow initiating the metastatic process. Thus, our results along with the findings that *CHL1* was a mutated candidate cancer-associated gene in colon cancer [1] suggested that this type of recognition receptors may indeed have dual roles in carcinogenesis. The mutations discovered in the extra-cellular part of *CHL1* could afford a therapeutic antibody to selectively treat patients [1]. This validates *CHL1* as a novel target for personalized immune intervention in cancers expressing mutated *CHL1*. New therapeutic small inhibitors directed at *CHL1* could be effective in restraining new tumor formation from dormant micrometastases.

Our results indicated that the *CHL1* gene could be important for the development of major human cancers, and also allowed to suggest a hypothesis on a probable dual role of *CHL1*, although only for three types of cancer (ovary, colon and breast) supportive data were thus far obtained. A frequent decrease of an expression level was prevalent for 11 of 19 tumor types and statistically significant for breast, colon, rectum, thyroid, kidney and small intestine cancer.

Our data also supported the role of *CHL1* as a potentially novel biomarker in the early pathogenesis of two major histological types of renal cancer both CC-RCC and pRCC. Results derived with 7 RCC cell lines suggested them as a potential model system for study of methylation role in *CHL1* silencing.

## Materials and Methods

### Cancer profiling arrays analysis

Cancer Profiling Arrays I and II (154 and 241 samples respectively, overall 19 different types of cancers namely, breast, kidney, rectum, colon, stomach, skin, thyroid, small intestine, bladder, vulva, pancreas, prostate, cervix, testis, lung, ovary, uterus, liver, trachea) purchased from BD Biosciences Clontech (Palo Alto, CA), were used to analyze the expression of the *CHL1* gene in normal and tumor tissues. Full sample information for Array I and II is presented in Clontech Catalog: No. 7841-1 and No. 631777 respectively (see “Arrays Information S1”).

We analyzed only informative samples with clear ratio of normal-tumor spots intensity. The information for samples of the Cancer Profiling Array I is presented below.

**1. Breast.** Most of the tumors are infiltrating ductal (DC), intraductal (IC) and lobular (LC) carcinomas. Stage I: 2Q, 2R, 2S, 2T, 2U, 4S, 4D, 4F. Stage II: 2W, 4E, 4L, 4N. Stage III: 4A, 4B, 4H, 4J, 4M. Coordinates of 18 metastatic (m) tumors are 2B, 2C, 2D, 2E, 2H, 2I, 2J, 2M, 2N, 2P, 4H, 4J, 4L, 4O, 4P, 4R, 4S, 4U. Coordinates of metastases are 4G, 4I, 4K.

**2. Uterus.** Most of the tumors are adenocarcinomas (ADC). Stage I: 8C, 8F, 8H, 8I, 8J, 8K, 8L, 8M, 8N, 8O, 8P, 8Q, 8R, 8S, 8U, 8X, 8Y, 8BB, 8CC, 8DD, 8EE, 8FF, 10A, 10B, 10C. Stage II: 8T. Coordinates of metastatic tumors 8W and 8AA, Stage III. Coordinates of metastases are 8V, 8Z.

**3. Colon.** All tumors are ADC. Stage I: 14L. Stage II: 14M, 14P, 14Q, 14AA. Stage III: 14S, 14U, 14V, 14Y, 14BB. Other samples have no information about the Stage. Coordinates of 9 metastatic tumors are 14E, 14N, 14P, 14U, 14W, 14Y, 14CC, 16A, 16C. Coordinates of metastases are 14O, 14T, 14V, 14X.

**4. Stomach.** Most of the tumors are ADC. There is no information about the Stage. Coordinates of 11 metastatic tumors are 20A, 20B, 20E, 20F, 20H, 20I, 20K, 20S, 20T, 20V, 20X.

**5. Ovary.** Stage I: 24B, 24D, 24E. Stage II: 24F. Stage III: 24A, 24G, 24H, 24J, 24L. Most tumors are ADC. Coordinates of metastatic tumors are 24J, 24L, 24M, 24N. Coordinates of metastases are 24I, 24K.

**6. Cervix.** 24X (adenosquamous carcinoma).

**7. Lung.** Stage I: 28E (squamous cell carcinoma, SCC), 28F (carcinoid), 28H (SCC), 28I (ADC), 28J (ADC), 28K (SCC), 28L (bronchiolo-alveolar adenocarcinoma, BAC), 28M (SCC), 28N (ADC). Unknown Stage: 28A (SCC), 28B (BAC), 28C (SCC), 28D (SCC), 28G (SCC), 28O (m, ADC), 28P (m, BAC), 28Q (SCC), 28R (carcinoid), 28S (ASC), 28T (SCC), 28U (SCC).

**8. Kidney.** Stage III: 32D (carcinoid). Unknown Stage: 32A (clear cell renal cell carcinoma, CC-RCC), 32B (RCC), 32C (RCC), 32E (RCC), 32F (transitional cell carcinoma), 32G (RCC), 32H (m, RCC), 32I (oncocytoma), 32J (RCC), 32K (RCC), 32L (m, RCC), 32M (RCC), 32N (m, RCC), 32O (RCC), 32P (RCC), 32Q (RCC), 32R (RCC), 32S (RCC), 32T (RCC).

**9. Rectum.** All tumors are ADC. Stage I: 36G. Stage II: 36J, 36F. Stage III: 36C, 36H, 36I, 36L. Coordinates of 6 metastatic tumors are 36B, 36E, 36L, 36M, 36Q, 36R. Coordinate of metastasis is 36K.

**10. Small intestine.** 36Y (m, ADC), 36Z (ADC).

**11. Thyroid gland.** All tumors are papillary ADC, Stage II: 40D. Stage III: 40C, 40E.

**12. Prostate.** All tumors are ADC. Stage I: 40M.

**13. Pancreas.** Unknown Stage: 40U (ADC).

The information for samples of the Cancer Profiling Array II is shown below.

**1. Breast.** Stage I: 6E (DC), 6G (mucinous ADC), 6M (DC). Stage II: 6I (DC), 6K (m, DC), 6L (DC), 6N (DC). Stage III: 6F (m, DC), 6H (m, DC), 6J (m, LC).

**2. Uterus.** Stage I: 6T (ADC), 6Z (SCC), 6BB (ADC). Stage II: 6X (SCC), 6AA (ADC). Unknown Stage: 6V (SCC).

**3. Ovary.** Stage I: 10E (papillary serous cystadenoma), 10K (mucinous cystadenocarcinoma). Stage II: 10I (ADC), 10J (leiomyoma). Stage III: 10G (ADC), 10H (CC-ADC), 10M (serous surface papillary carcinoma), 10N (papillary serous cystadenoma). Stage IV: 10L (ADC). Unknown Stage: 10F (leiomyoma).

**4. Cervix.** Stage I: 10Z (ADC), 10AA (SCC), 10BB (SCC). Stage II: 10V (SCC). Stage III: 10S (m, SCC).

**5. Colon.** Stage I: 14E (tubulovillous adenoma, other tumors are ADC), 14F. Stage II: 14L. Stage III: 14H (m), 14I (m), 14J, 14K (m), 14M, 14N (m). Stage IV: 14G (m).

**6. Rectum.** All tumors are ADC. Stage II: 14X, 14Y, 14Z. Stage III: 14T, 14V (m), 14W (m), 14AA (m), 14BB (m). Stage IV: 14S (m), 14U (m).

**7. Stomach.** Most of the tumors are ADC. Stage II: 18E. Stage III: 18M (m). Stage IV: 18J (m), 18K (m). T3N1Mx: 18F (m).

**8. Thyroid gland.** Stage II: 18X (papillary adenocarcinoma, PAC), 18Y (PAC), 18AA (PAC), 18BB (follicular ADC). Stage III: 18S (m, ADC), 18W (m, PAC). Stage IV: 18T (m, medullary carcinoma), 18Z (m, PAC). T3N0M0: 18U (PAC).

**9. Lung.** Stage I: 22I (SCC), 22J (SCC), 22N (SCC). Stage II: 22L (m).

**10. Testis.** All tumors are seminomas. Stage I: 22V, 22X. Stage II: 22T. Stage III: 22BB (m). Stage IV: 22AA (m).

**11. Kidney.** Stage II: 26E (RCC), 26M (epithelial nephroblastoma). Stage III: 26H (RCC), 26I (transitional cell carcinoma, TCC), 26J (clear cell ADC), 26K (m, TCC). Stage IV: 26G (m, RCC), 26L (m, RCC). Unknown Stage: 26F (oxyphilic adenoma). T3aNxM0: 26N.

**12. Skin.** Most of the tumors are melanomas. Stage I: 26S, 26BB. Stage II: 26Y (SCC), 26Z (SCC). Stage III: 26T, 26U, 26W, 26AA. Stage IV: 26V. Unknown Stage: 26X.

**13. Bladder.** All the tumors are transitional cell carcinomas: Stage II: 30E, 30H. Stage III: 30F, 30I. Stage IV: 30G.

**14. Trachea.** T4N1M0: 30L (m); T4N0M0: 30M. T3N1M0: 30N (m).

**15. Small intestine.** Most of the tumors are ADC. Stage II: 30T, 30V. Stage III: 30S (m), 30U, 30X (m). Stage IV: 30Y (m). Unknown Stage: 30W (carcinoid).

**16. Vulva.** All tumors are SCC. Stage II: 34E, 34F, 34G, 34H. T2aN1M0: 34I (m).

**17. Liver.** T3N0M0: 34L. T2N0M0: 34M. T2N0M0: 34N.

**18. Pancreas.** Stage I: 34S (ADC), 34T (ADC). Stage II: 34V (SCC). Stage IV: 34W (m, ADC). T3NxM0: 34Y (cancer of caput of pancreas). Unknown Stage: 34X (carcinoid).

**19. Prostate.** All tumors are ADC. Stage I: 38E. Unknown Stage: 38F, 38G, 38H.

Totally, there were 90 metastatic samples (with lymph node metastases) and 12 metastases at distant sites.

The arrays were hybridized with specific *CHL1* probes labeled with ^32^P-α-deoxycytidine triphosphate according to manufacturer's protocol (Clontech). The images were obtained using the Packard Cyclone Storage Phosphor System (PerkinElmer, Shelton, CT).

### Tissues specimens and cell lines for RT-qPCR analysis

We analyzed cDNA samples isolated from 30 NSCLC specimens including 16 SCC and 14 ADC; 39 renal cancer specimens including 30 CC-RCC and 9 papillary renal cell carcinomas (pRCC) and 23 breast cancer primary tumors including 19 ductal BC, one lobular BC and 3 mixed lobular-ductal BC. Accordingly, we used 29, 39 and 23 samples from adjacent morphologically normal lung, renal and breast tissues as controls. Six samples of normal lung tissues from healthy donors were used as additional controls. Paired tumor and non-cancerous (normal) matched tissues were received from Blokhin Cancer Research Center, Russian Academy of Medical Sciences. All tumors were classified according to the International TNM Classification system [49], [50]. These samples were obtained from patients after surgical resection of primary lung, renal and breast cancer prior radiation or chemotherapy and stored in liquid nitrogen. The diagnosis was verified by histopathology and only samples containing 70–80% or more tumor cells were used in the study. The samples were collected in accordance to the guidelines issued by the Ethics Committee of Blokhin Cancer Research Center, Russian Academy of Medical Sciences (Moscow). All patients gave written informed consent that is available upon request. The Ethics Committee of Blokhin Cancer Research Center, Russian Academy of Medical Sciences specifically approved this study. The study was done in accordance with the principles outlined in the Declaration of Helsinki.

Clear cell renal cancer cell lines TK10, TK164, KRC/Y, KH39, HN4, Caki1and Caki2 from MTC collection were also analyzed in the study. Mix of six samples of normal lung tissues from healthy donors were used as controls.

### RNA and cDNA preparation for RT-qPCR

Total RNA was isolated from tumor and matched normal tissues, renal cancer cell lines using RNeasy Mini Kit (Qiagen, Netherlands) according to the manufacturer's instructions. Purified RNA was quantified using NanoDrop® ND-1000 spectrophotometer (NanoDrop Technologies Inc., USA), and the quality was determined by Bioanalyzer 2100 (Agilent Technologies, USA). All RNA samples were treated with DNase I and cDNA was synthesized using MMLV reverse transcriptase and random hexamers according to standard manufacturer's protocol (Fermentas, Lithuania).

### Analysis of CHL1 expression by RT-qPCR

RT-qPCR was performed essentially as described previously [51] in total volume of 25 µl in triplicate. The sequences of primers (F and R) and TaqMan probes (Z) are shown in Table 5. Final concentrations of primers and probes for target and reference genes were as follow: *CHL1* primers – 300 nM, probe – 400 nM; *GAPDH* primers – 300 nM, probe – 150 nM, *RPN1* primers – 300 nM, probe – 150 nM, *GUSB* primers – 300 nM, probe – 250 nM, *B2M* primers and probe – 300 nM.

**Table 5:** Primers and probes sequences for *CHL1* and reference genes for RT-qPCR.

Symbol/RefSeq ID	Gene name	Primers (F, R) and probe (Z) sequences5′→3′
*CHL1* (*CALL*)NM 006614.2	Close homolog of L1 (cell adhesion L1 like)	F: GAACTATCCTTGCCAATGCCAATATR: TTCTGCCAGGACACGACTGCZ: AAGAAAGCACTGTACCCAACCACTGTAGCG
*RPN1*NM_002950.3	Ribophorin I	F: CACCCTCAACAGTGGCAAGAAGR: TGCATTTCGCTCACTCTGTCGZ: CCCTCTGTCTTCAGCCTGGACTGC
*GUSB*NM_000181,2	Glucuronidase, betaβ-D-glucuronidase	F: GATGGAAGAAGTGGTGCGTAGGR: TTAGAGTTGCTCACAAAGGTCACAGZ: CGTCCCACCTAGAATCTGCTGGCTACTACTT
*B2M*NM_004048.2	Beta-2-microglobulin	F: ATGAGTATGCCTGCCGTGTGR: AATTCATCCAATCCAAATGCGZ: ATCTTCAAACCTCCATGATGCTGCTTACAT
*GAPDH*NM _002046.3	Glyceraldehyde-3-phosphate dehydrogenase	F: GGAGTCAACGGATTTGGTCR: TGGGTGGAATCATATTGGAACATZ: CCTTCATTGACCTCAACTACATGGTTTACAT

The thermocycler conditions were 10 min at 95°C, then 45 two-step cycles 15 s at 95°C and 60 s at 60°C (ABI 7000 PRISM Sequence Detection System, Applied Biosystems). The sequences of the amplicons were verified by sequencing in a 3730 DNA Analyzer (Applied Biosystems).

RT-qPCR data were analyzed using the relative quantification method, or 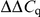-method [52], [53] based on target and reference gene, in tumor (T) and normal (N) samples comparison. Relative mRNA level (*R*) is calculated by formula: 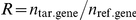, where *n* is mRNA copy number in tumor sample relative normal sample. 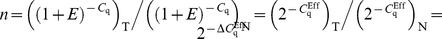. Here is *E* – reaction efficiency, 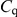 – quantification cycle, 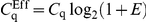, effective quantification cycle.

Finally, 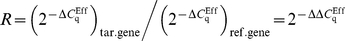.

The reaction efficiencies (*E*) were calculated as described [33] and their values were 85±9% (lung), 93±11% (breast), 87±10% (renal) for *CHL1*; 89±8% (lung) for *GAPDH*; 86±11% (breast) for *B2M*; 87±9% (renal) for *RPN1*; 81±9% (renal) for *GUSB* in tumor and normal samples. The level of mRNA expression change was calculated as 1/*R* and reflected the *n*-fold factor by which the mRNA content decreased or increased in the tumor compared to normal tissue.

All preliminary validation steps were performed according to Gene quantification resource (www.gene-quantification.de). As reference genes, we used *GAPDH* and *RPN1* for NSCLC, *B2M* for BC, and *GUSB* and *RPN1* for RCC. The relative inner variability between *GAPDH*, *B2M*, *GUSB* and *RPN1* mRNA levels was not higher than 2-fold in tumor (T) and normal (N) tissues, therefore 2-fold and more mRNA changes for *CHL1* were considered as significant for all types of studied tumors.

All calculations performed using our program ATG (Analysis of Transcription of Genes) compatible with Relative Quantification (RQ) software (Applied Biosystems). The program is designed for mathematical processing of RT-qPCR data in paired normal-tumor samples. The general features of AEGIS consist of estimating reaction efficiencies with three different methods (including calculating efficiency from kinetic curves) and taking it into account for further data analysis, using the relative comparative 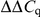
method [52] with several reference genes and estimating their variability. Also this program allows checking adjusted normal samples variability and using as paired normal sample for each tumor sample as one or several normal samples for all tumor samples.

### Statistical analysis

For validation of *CHL1* expression differences between normal and tumor samples and also between tumor samples with and without metastases we applied Fisher exact test and χ^2^ criterion. Nonparametric Wilcoxon test was used for analyzing RT-qPCR data to compare mRNA content differences of the *CHL1* gene and reference genes for the same samples. We used nonparametric Kruskal-Wallis and Mann-Whitney tests for the rank order differences between average relative levels of mRNA in different groups of samples. *P*-values<0.05 were considered statistically significant. All statistical procedures were performed using BioStat software [54].

## Supporting Information

Arrays Information S1 This archive contains detailed information about localization and clinical characteristics of all the tumors (histological type, tumor size, stage, presence of metastases etc.).
(DOC)
