# Supplementary figures and images for: Differential Expression of CHL1 Gene during Development of Major Human Cancers
Source: PLoS One. 2011 Mar 7;6(3):e15612. doi: 10.1371/journal.pone.0015612 (PMC3049765; doi:10.1371/journal.pone.0015612)

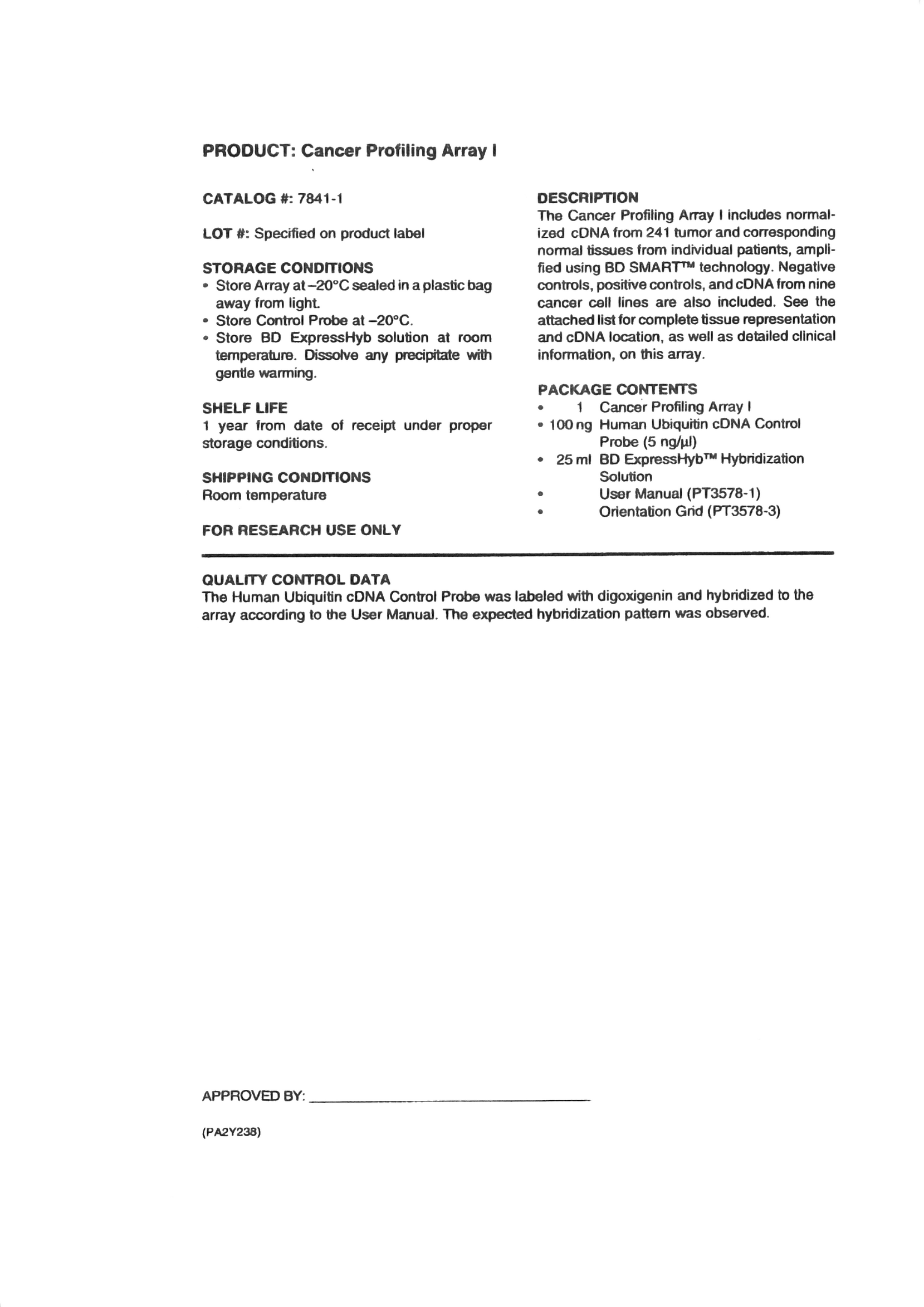


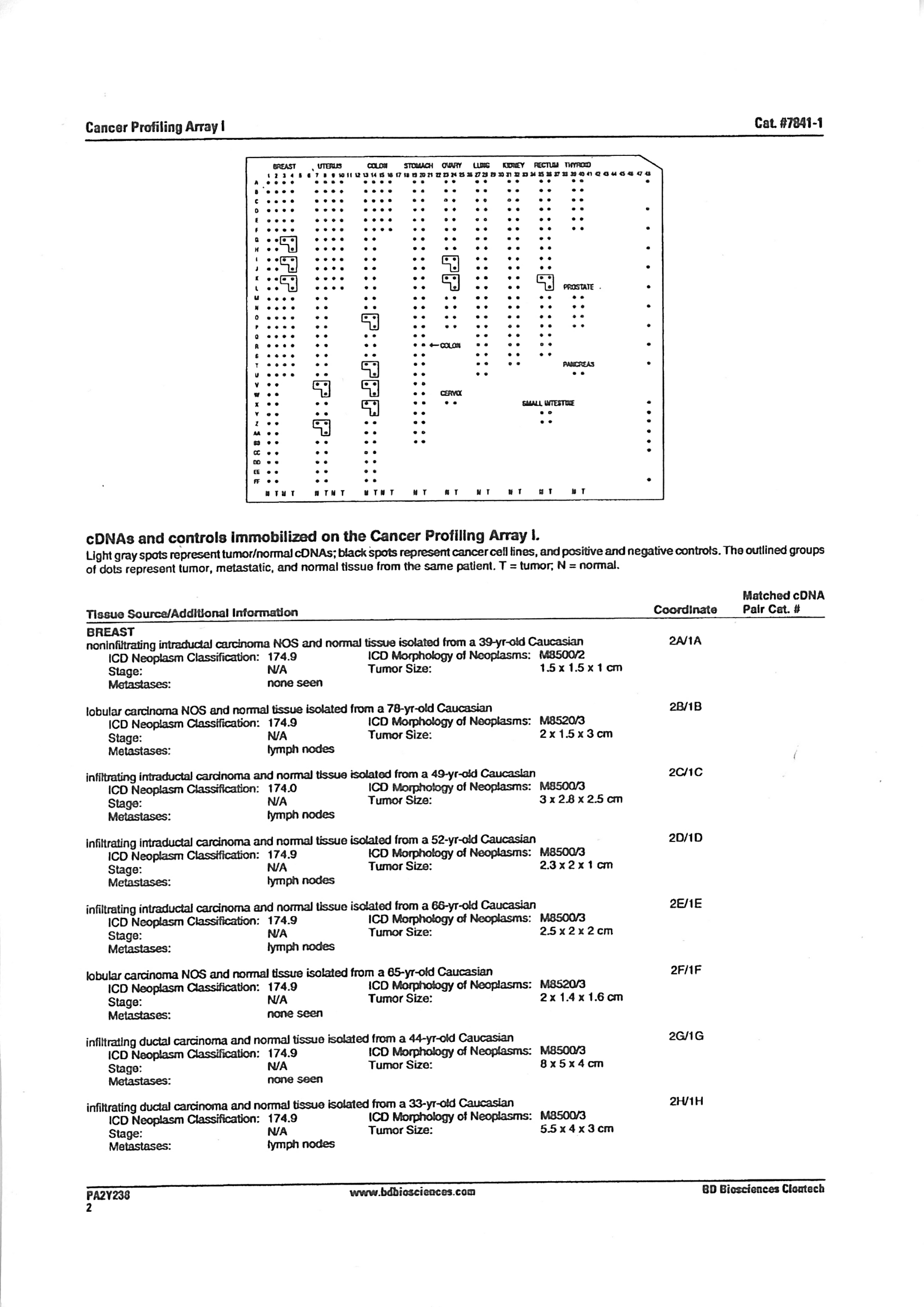


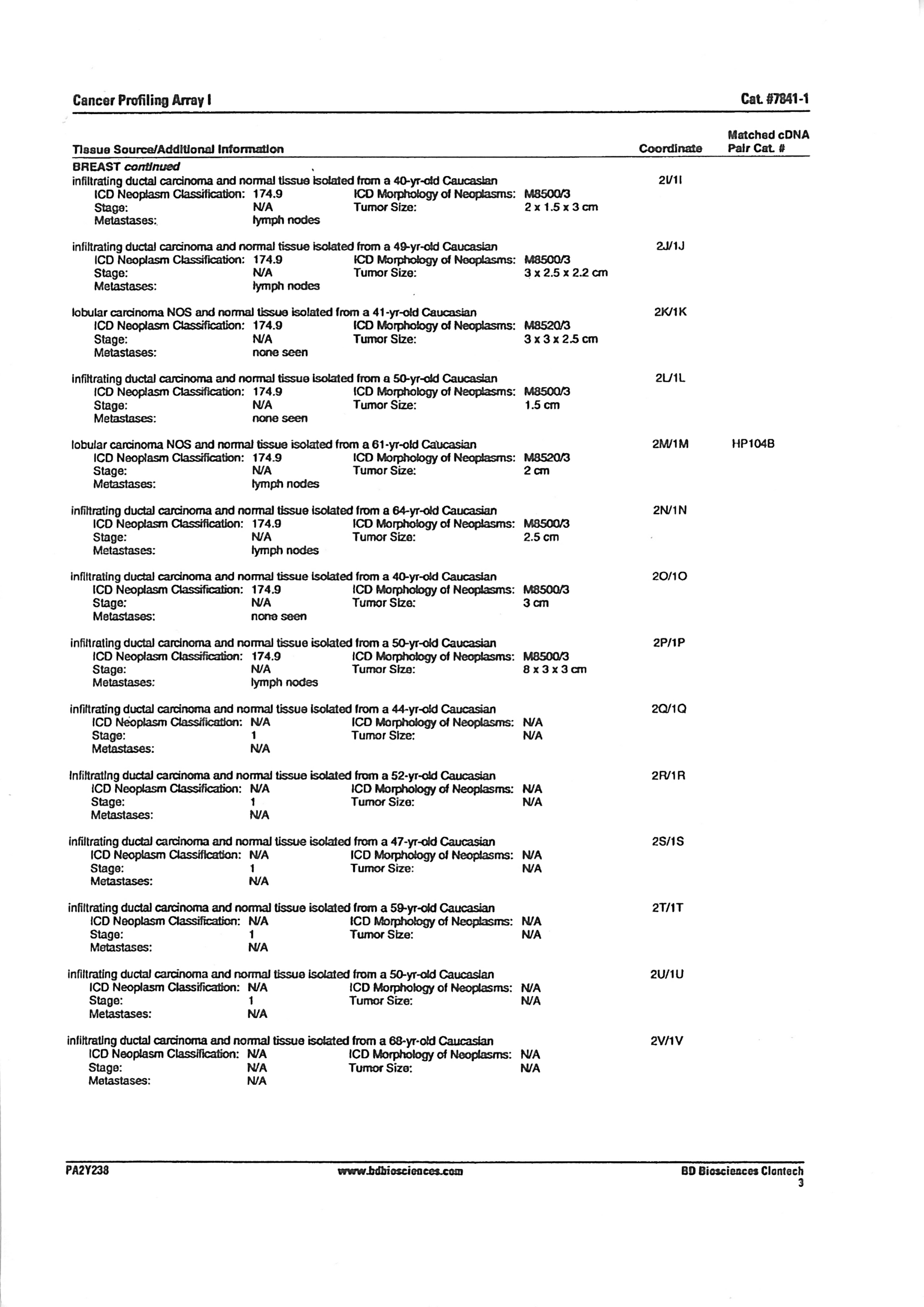


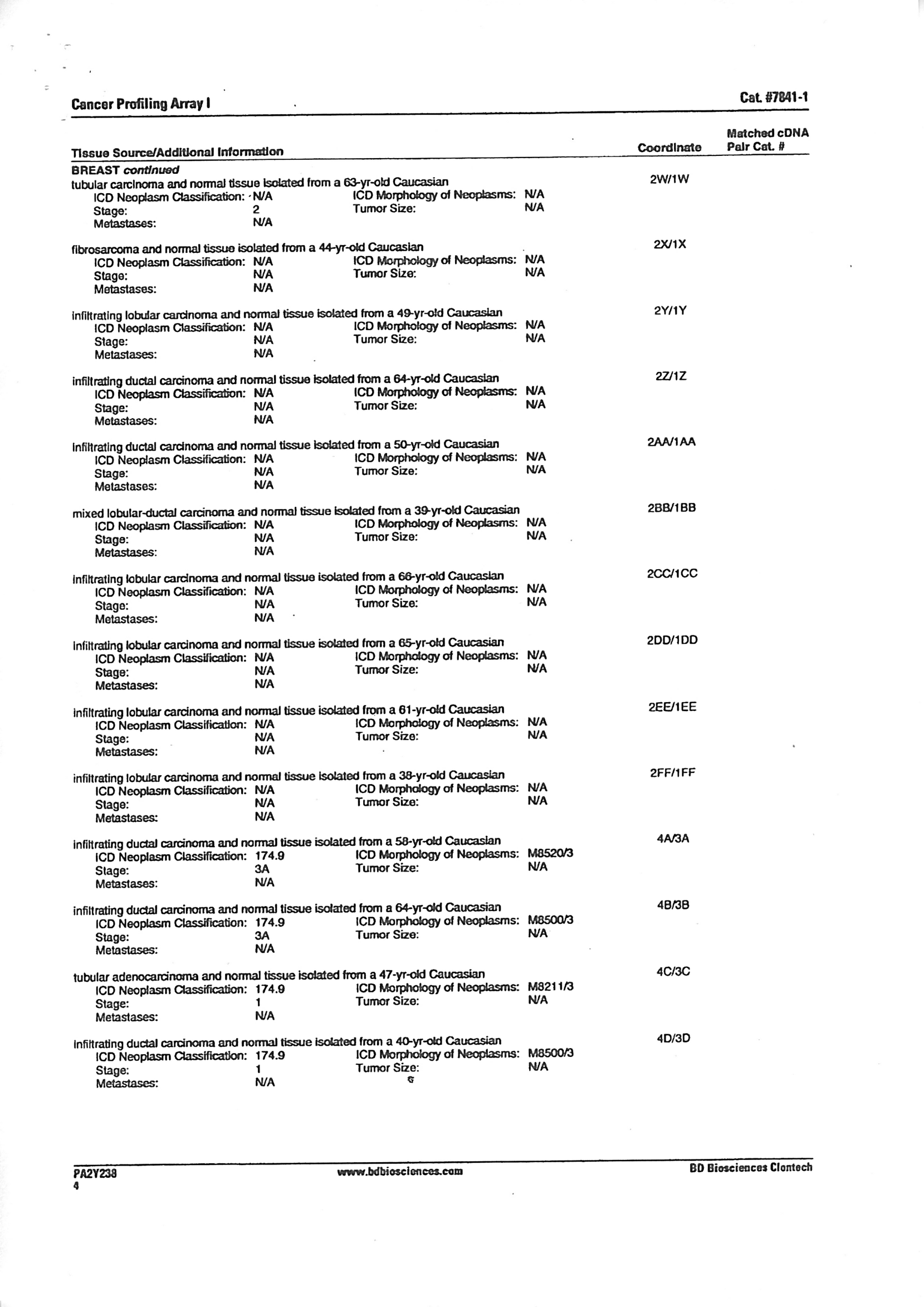


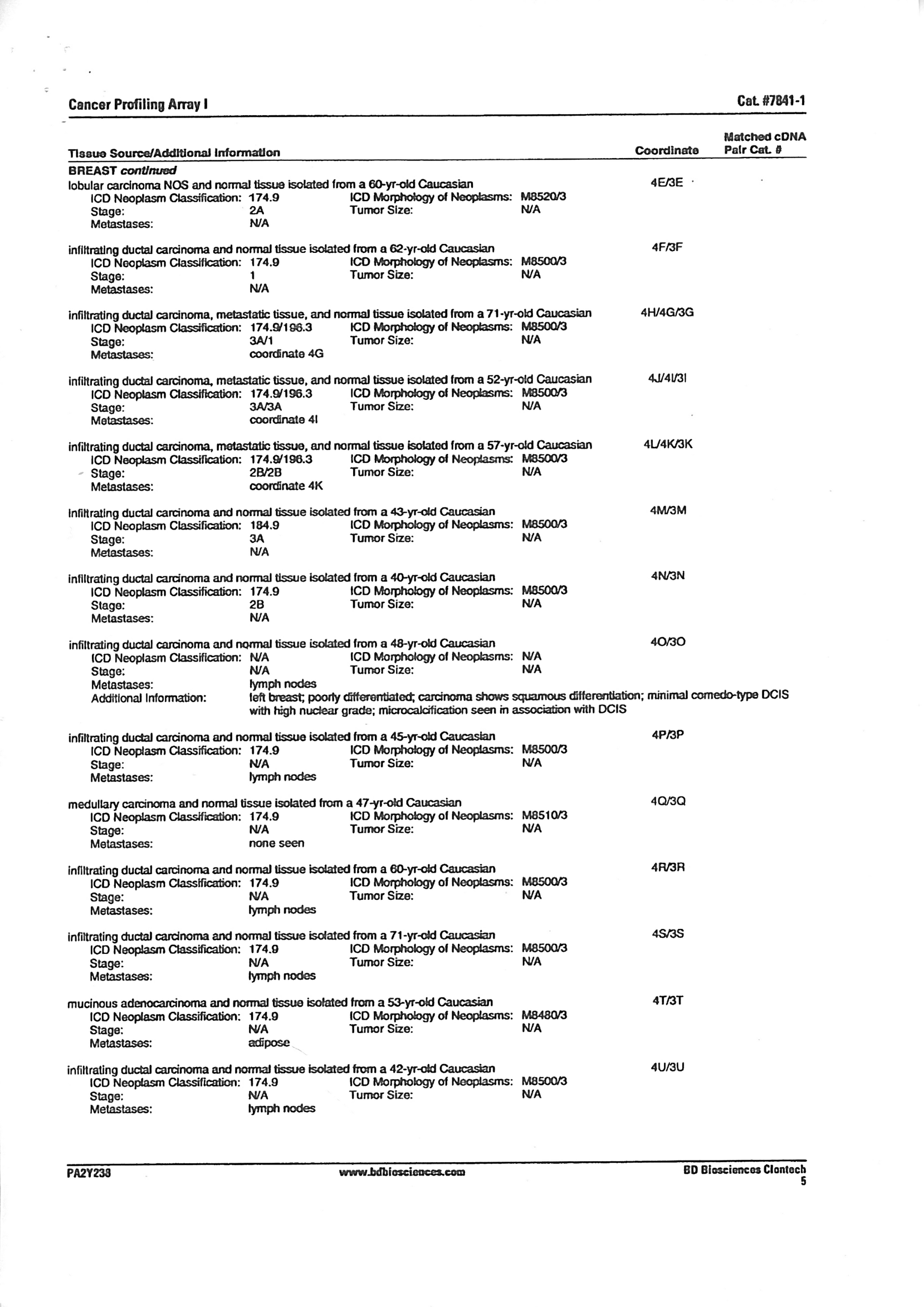


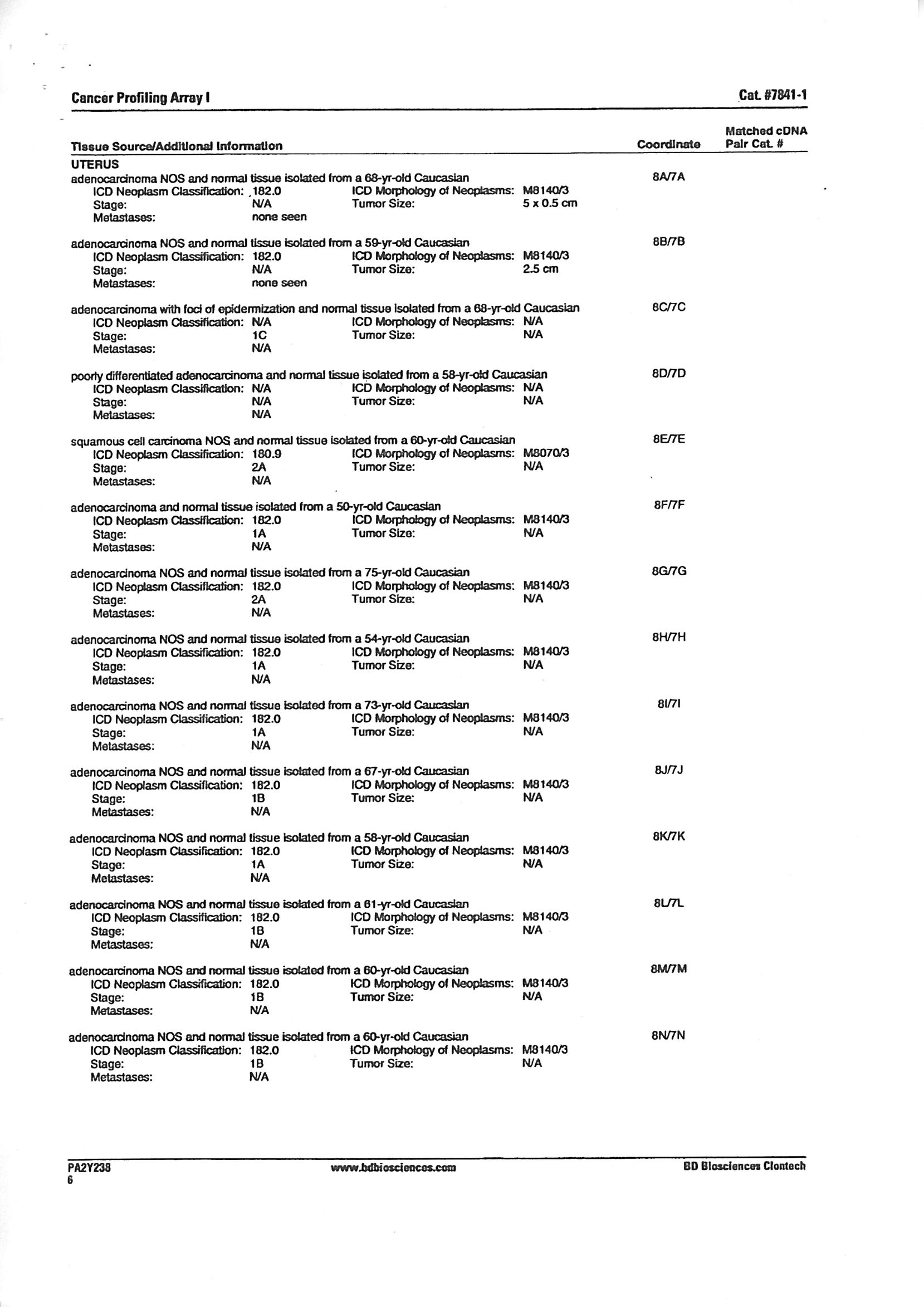


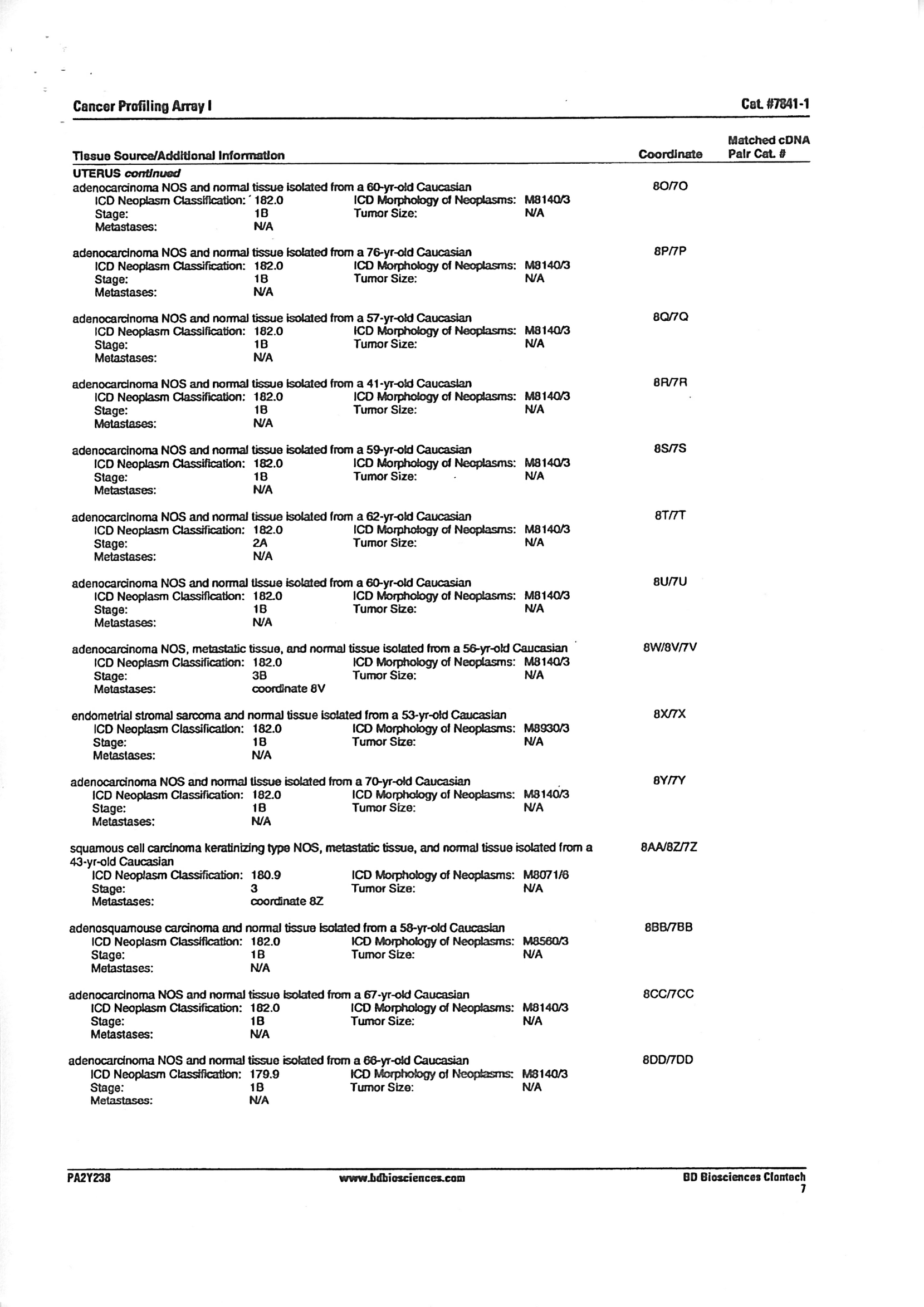


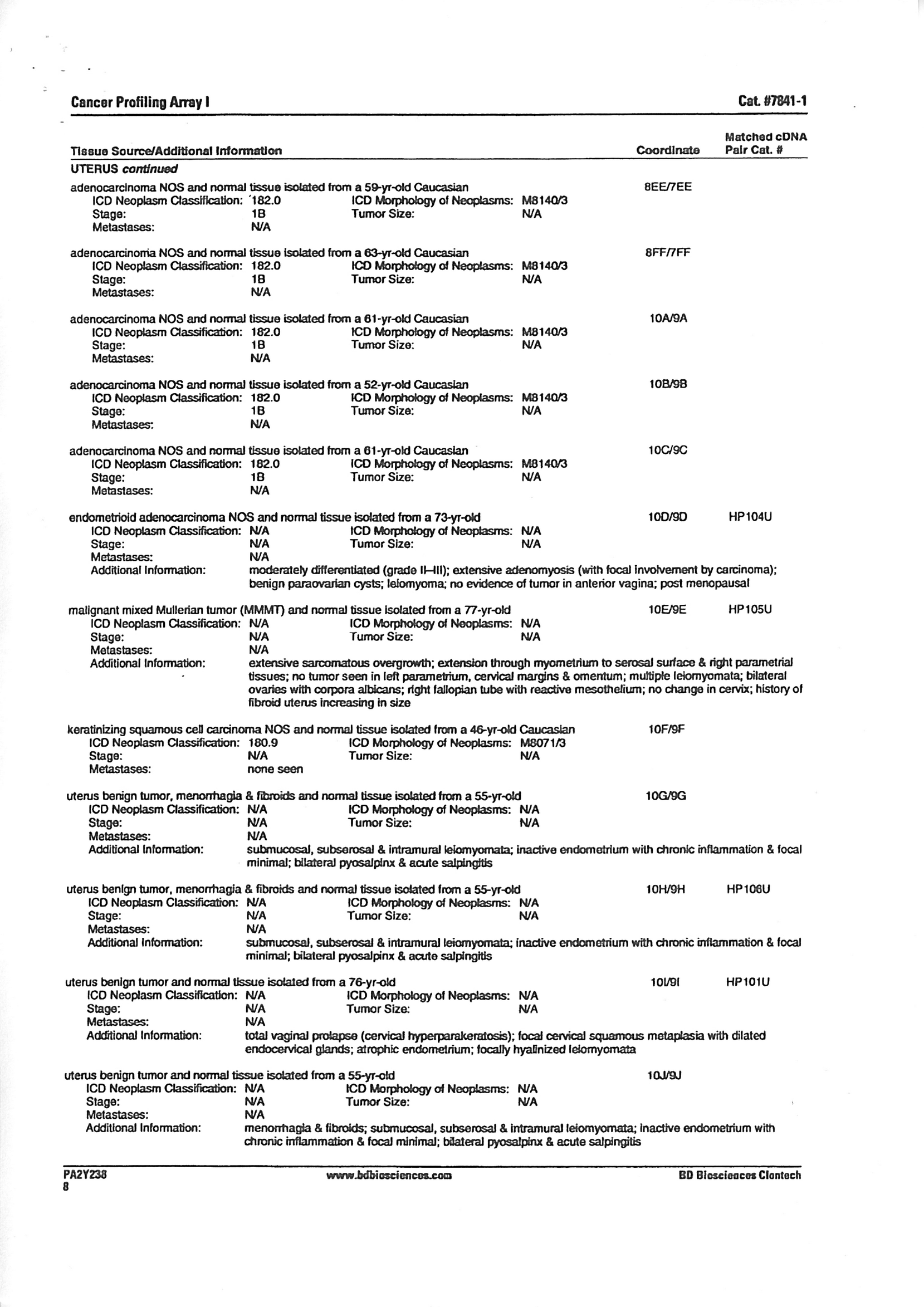


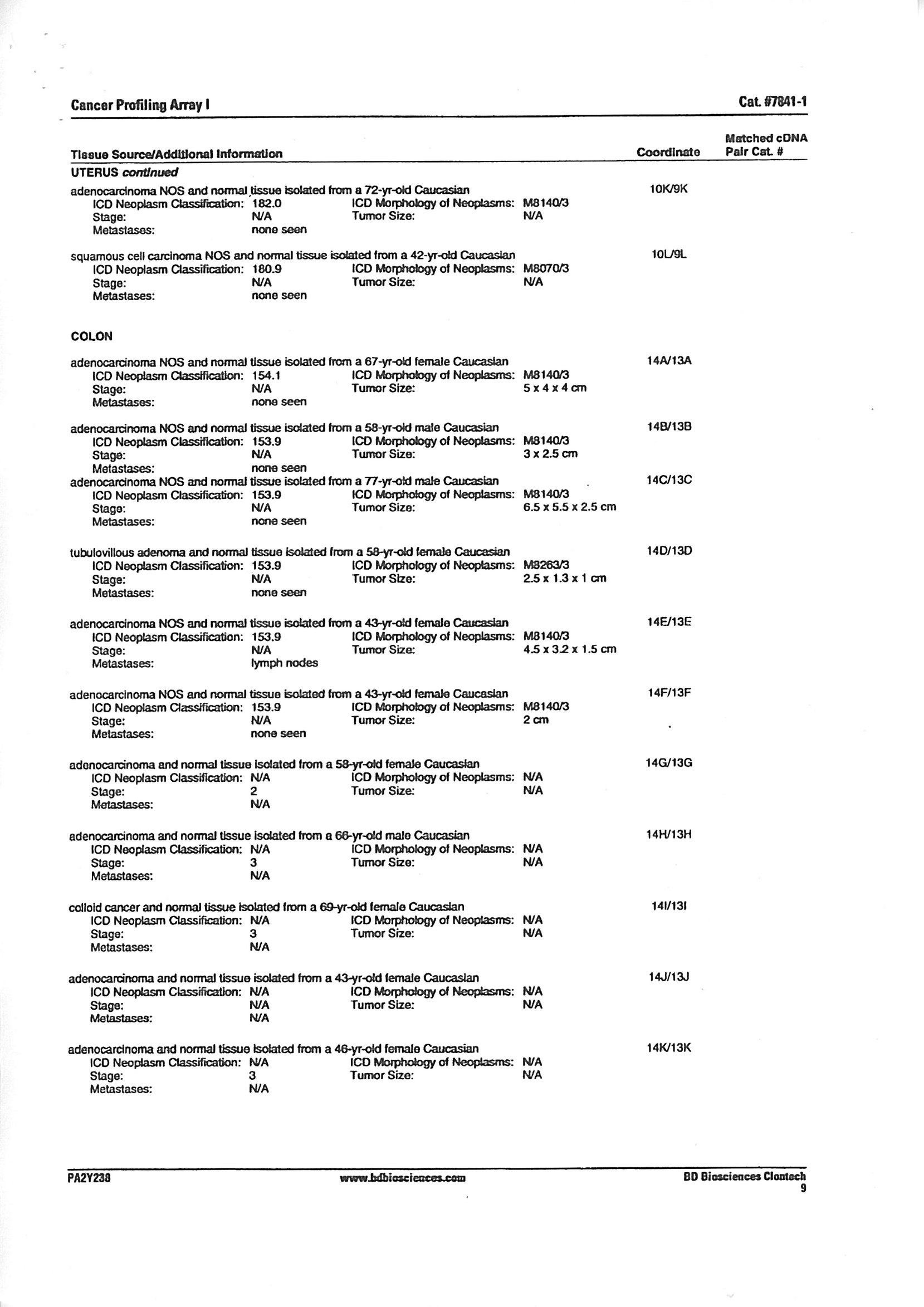


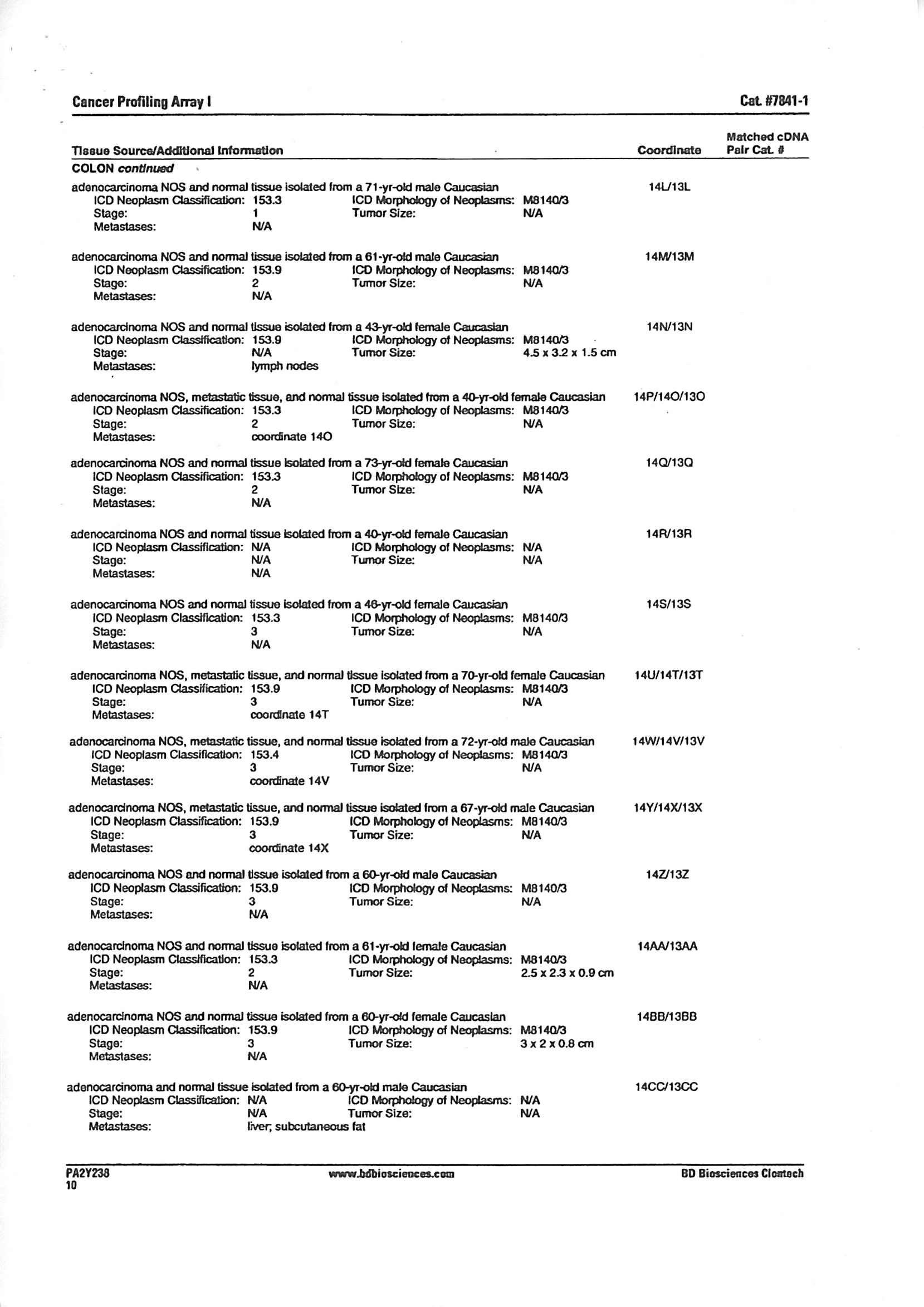


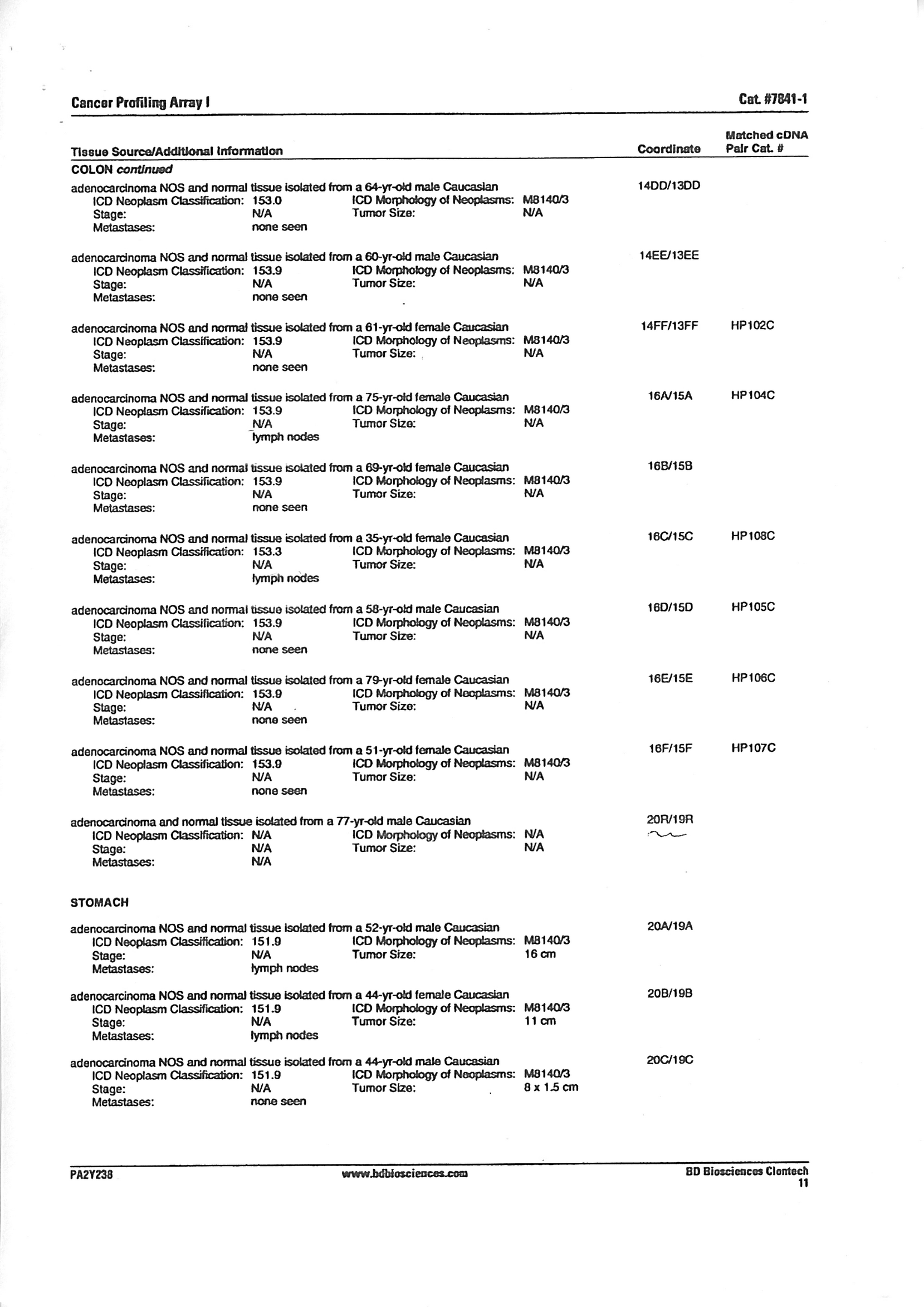


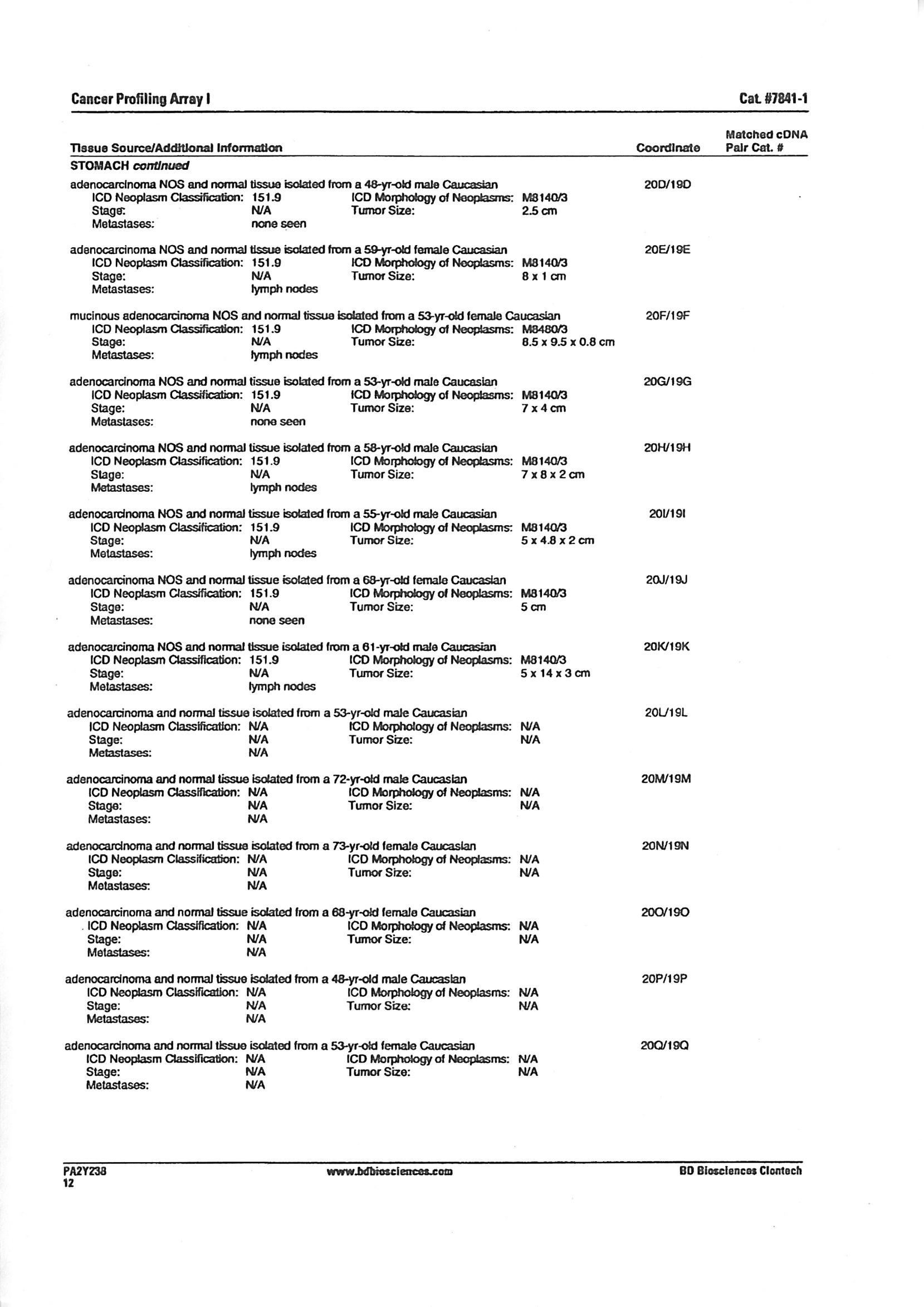


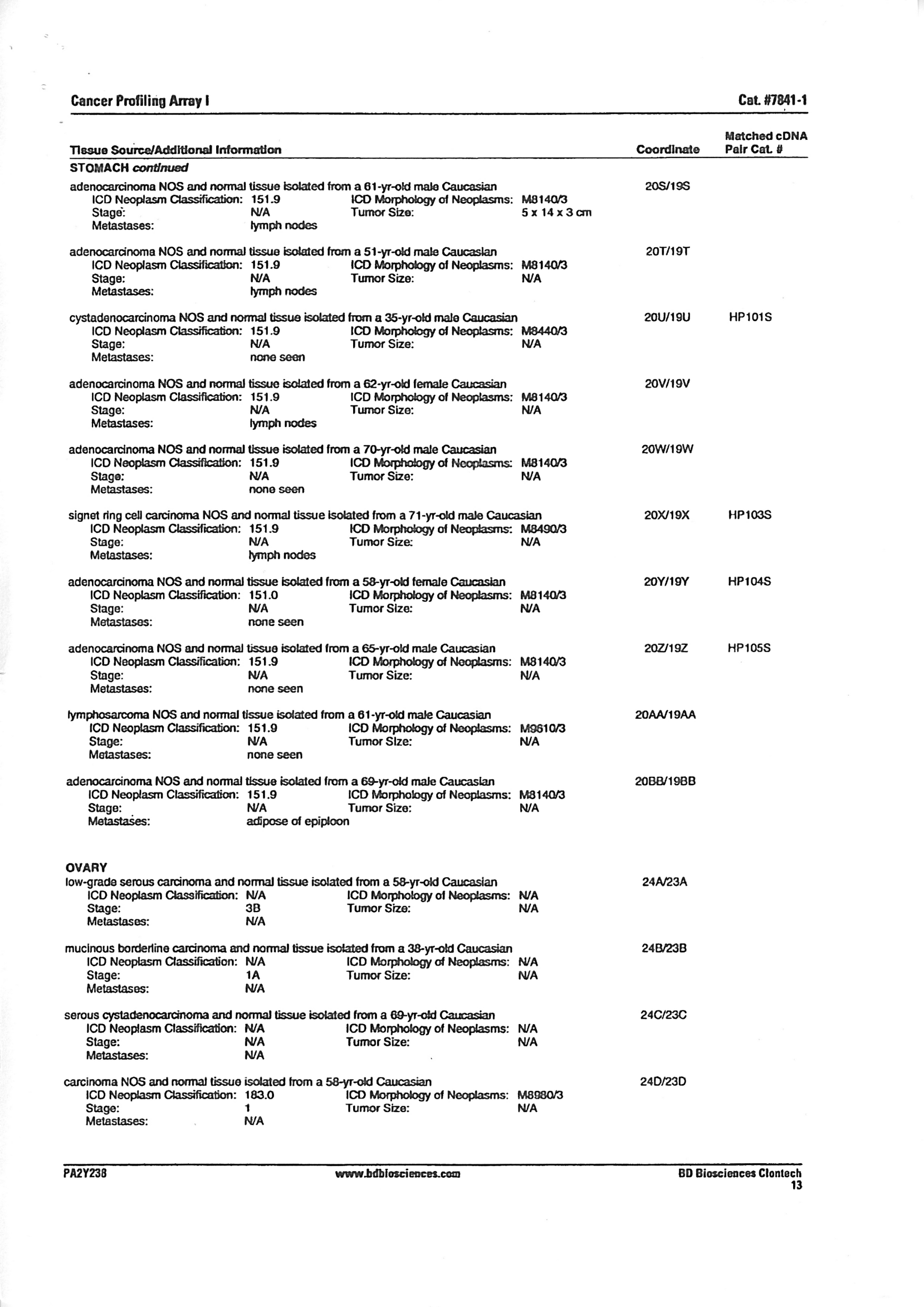


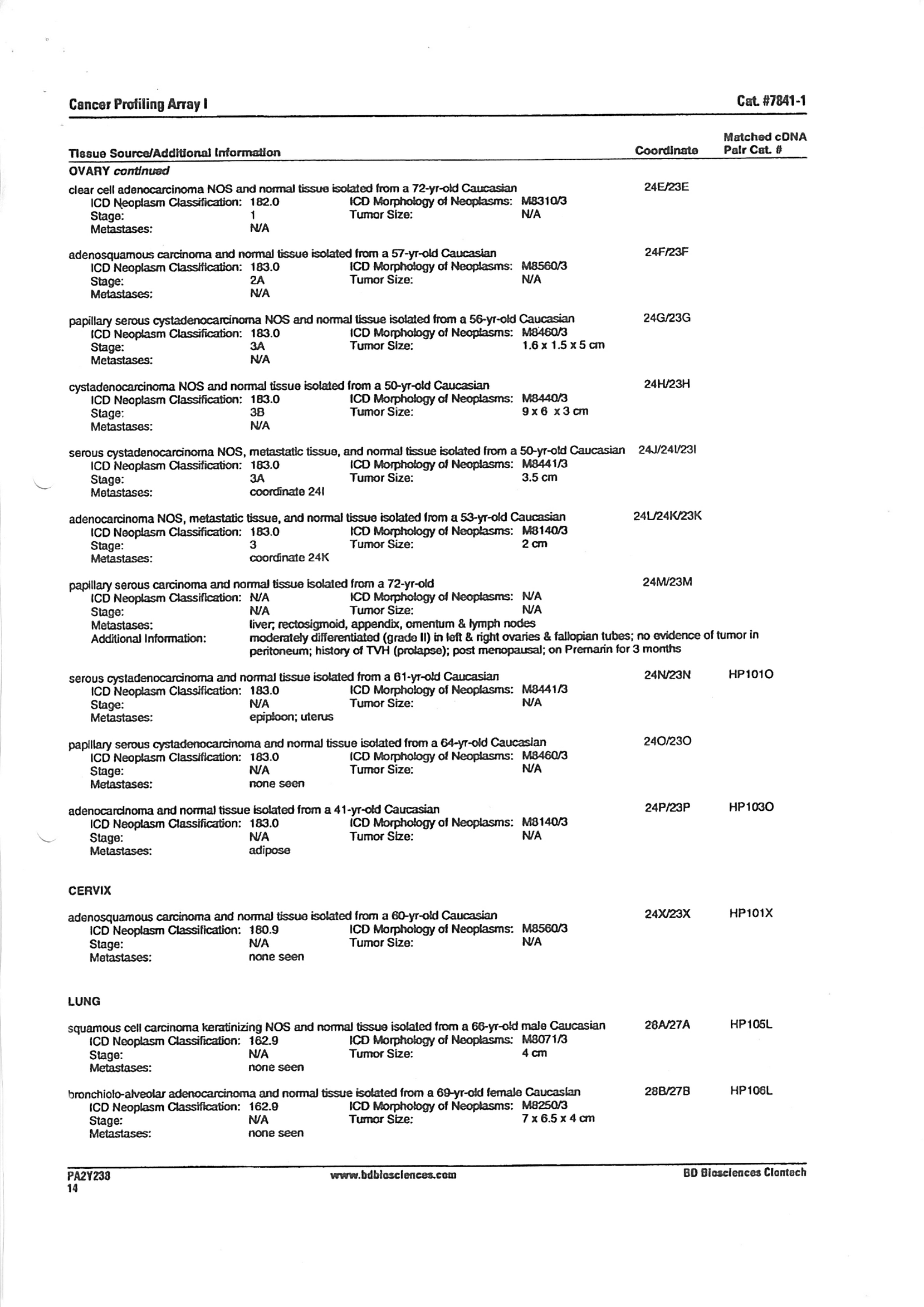


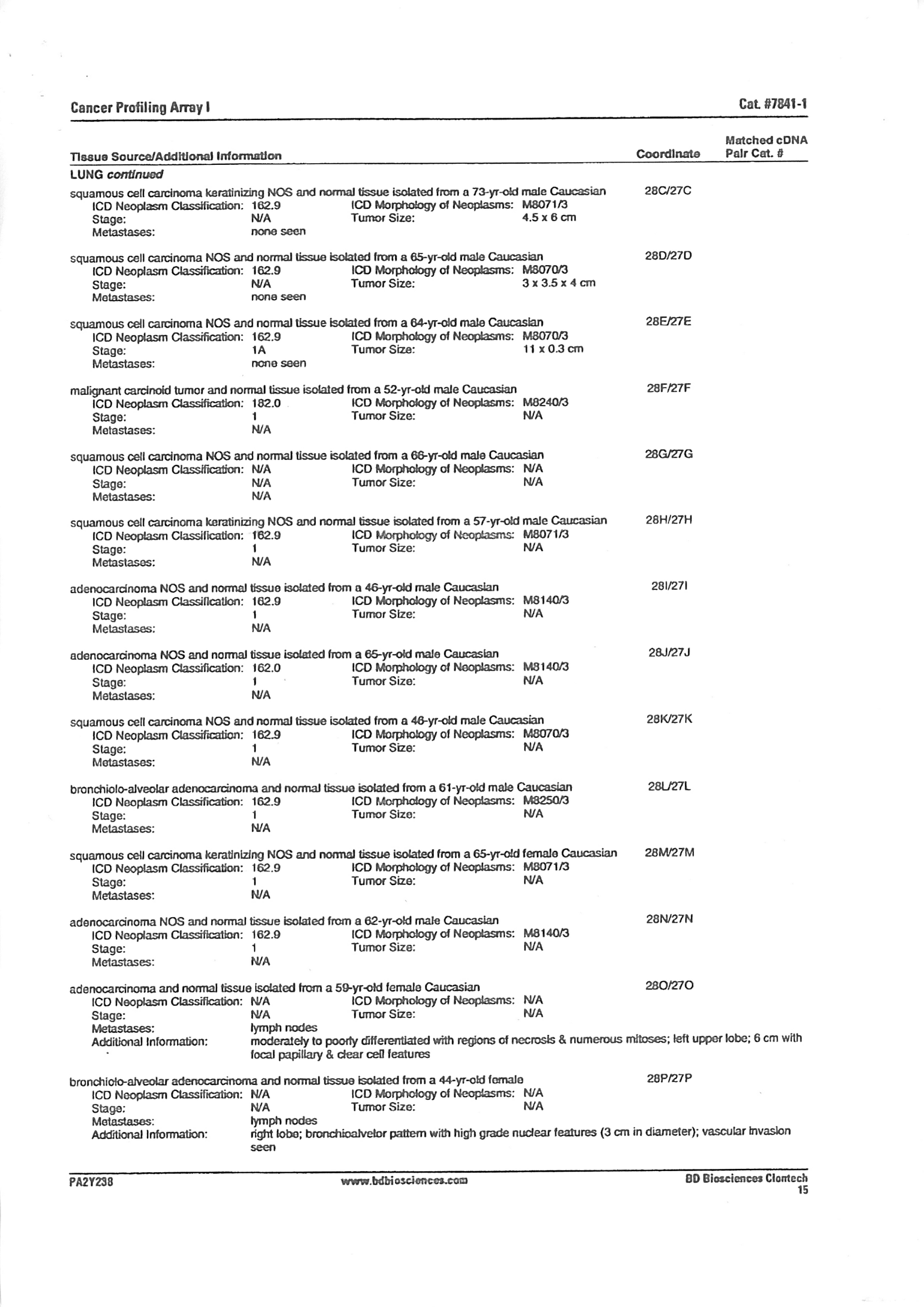


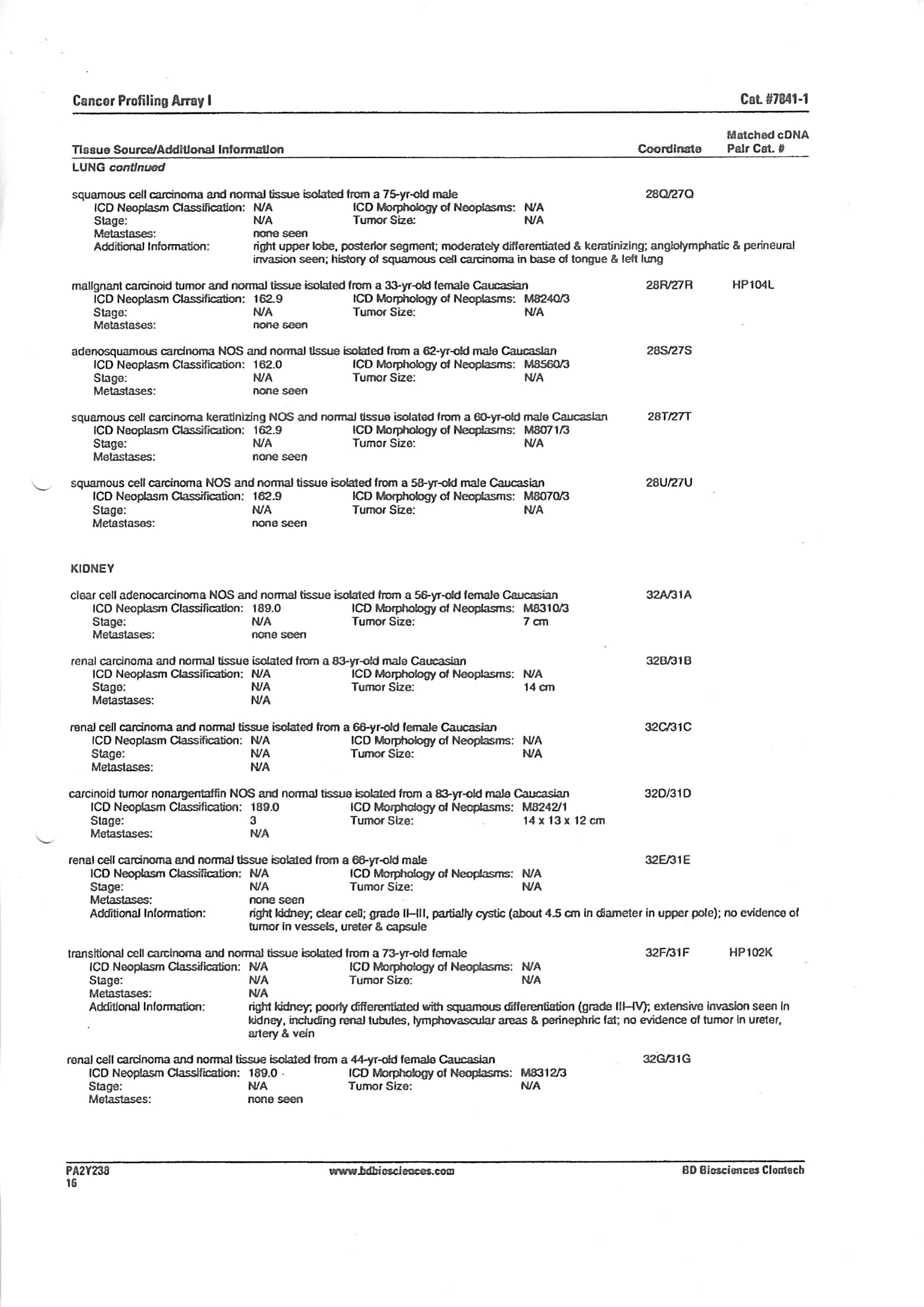


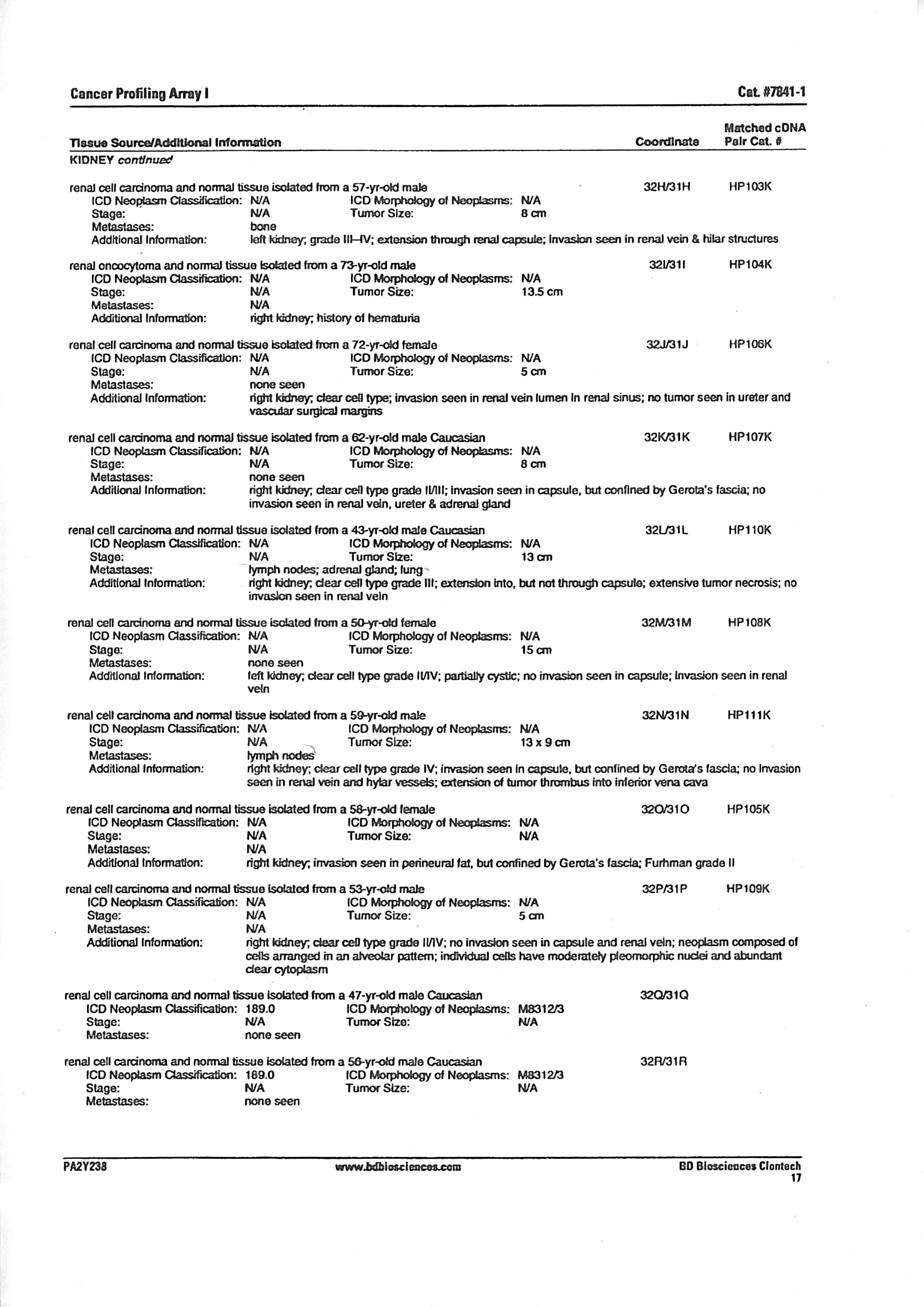


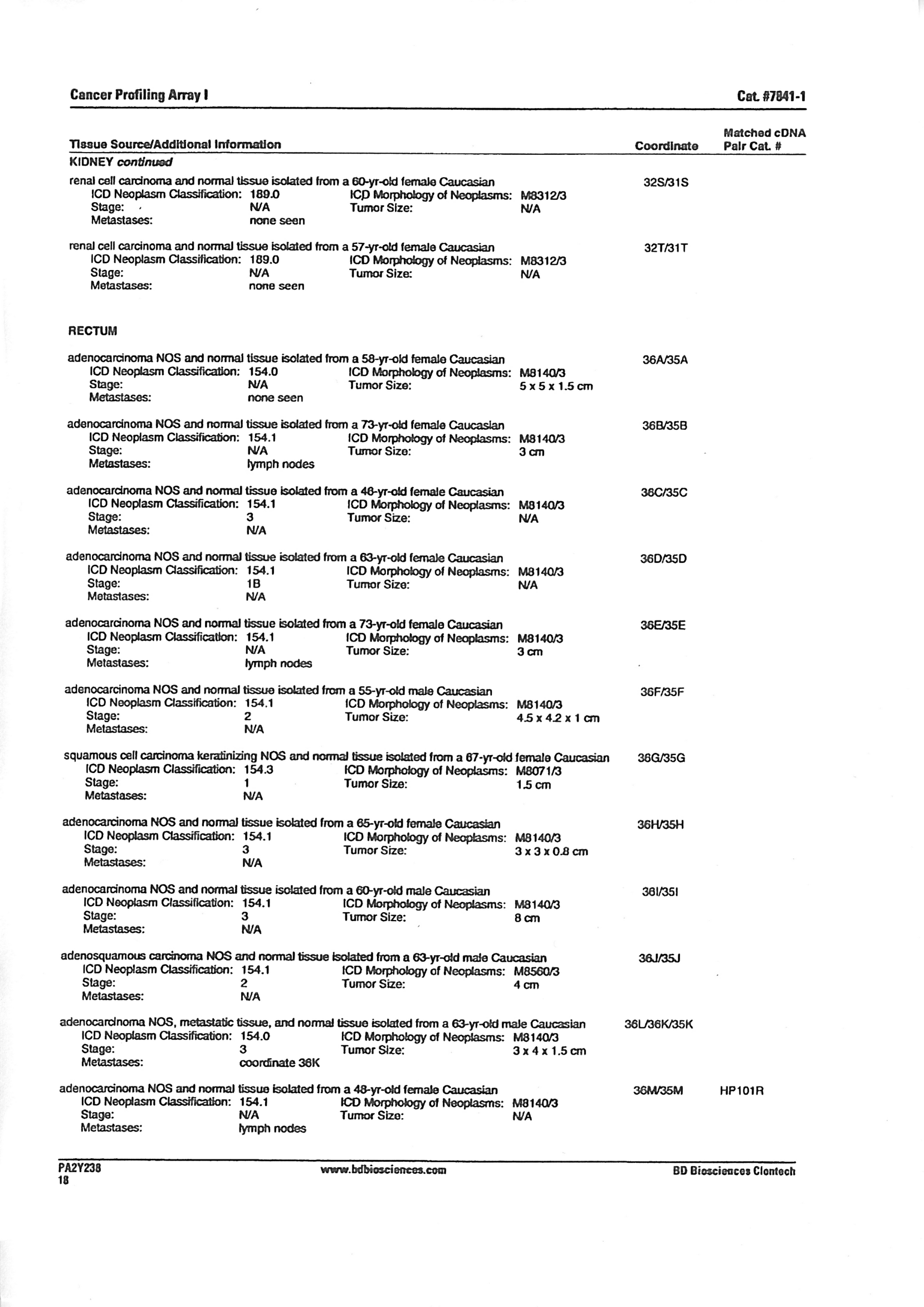


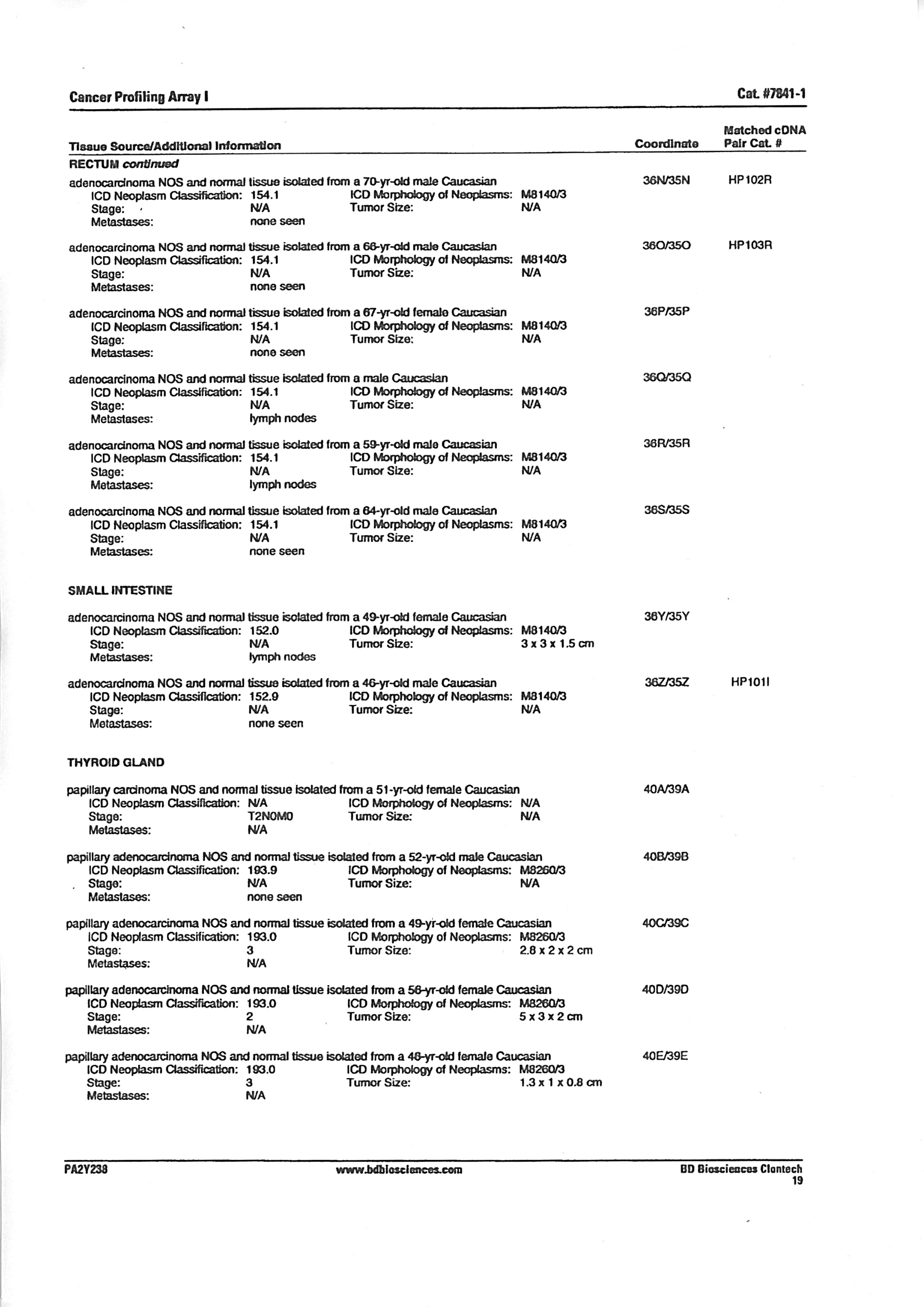


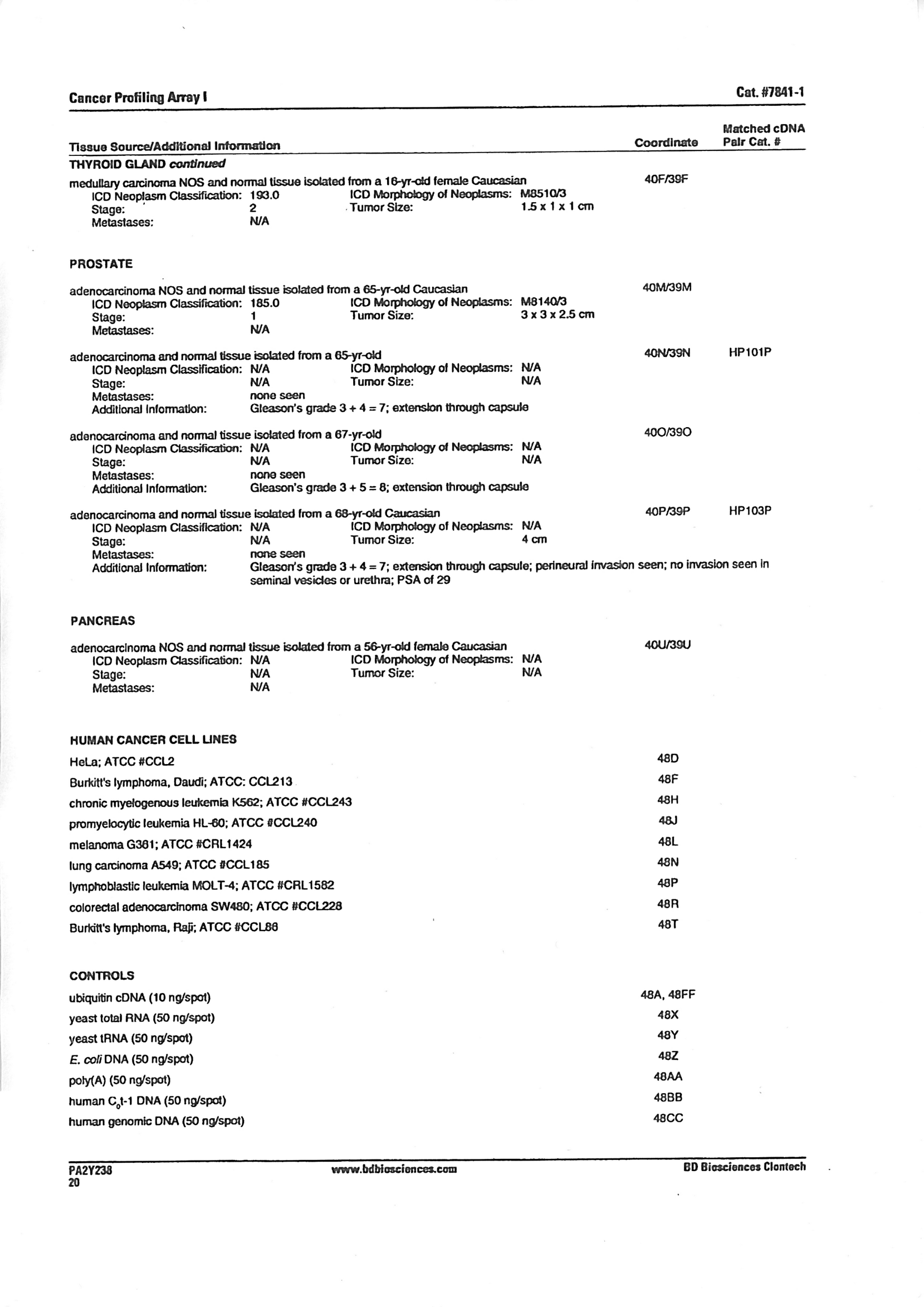


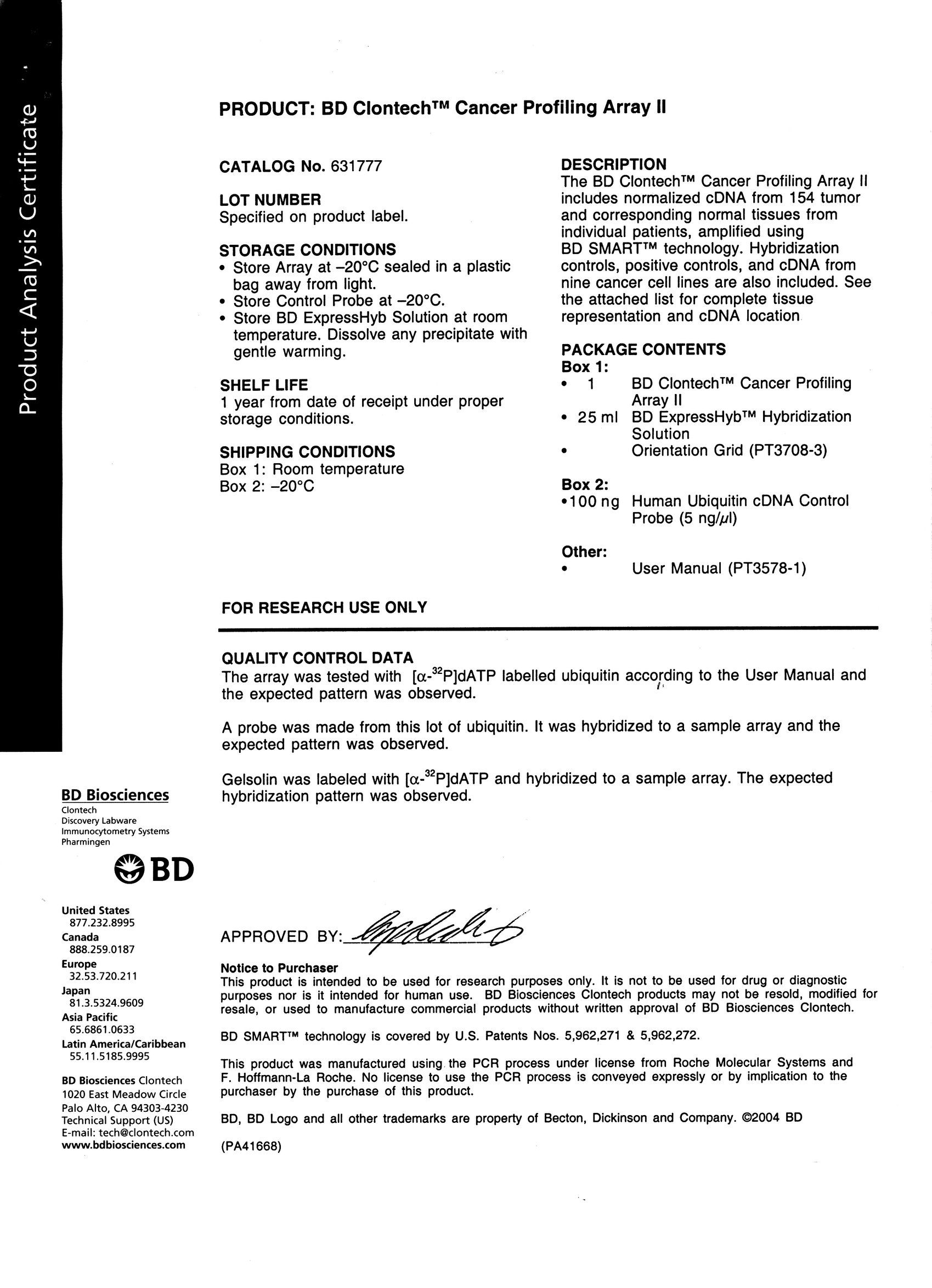


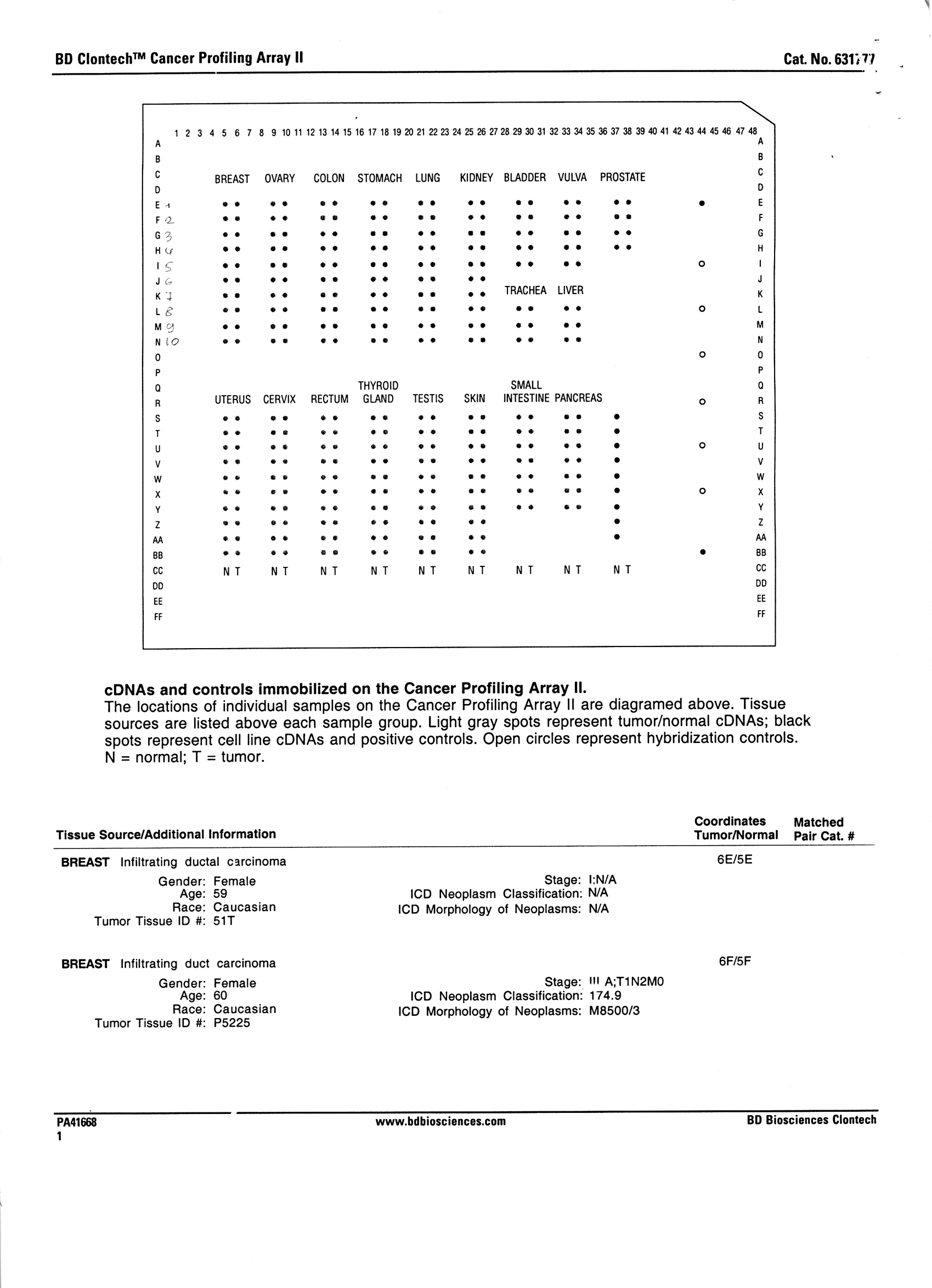


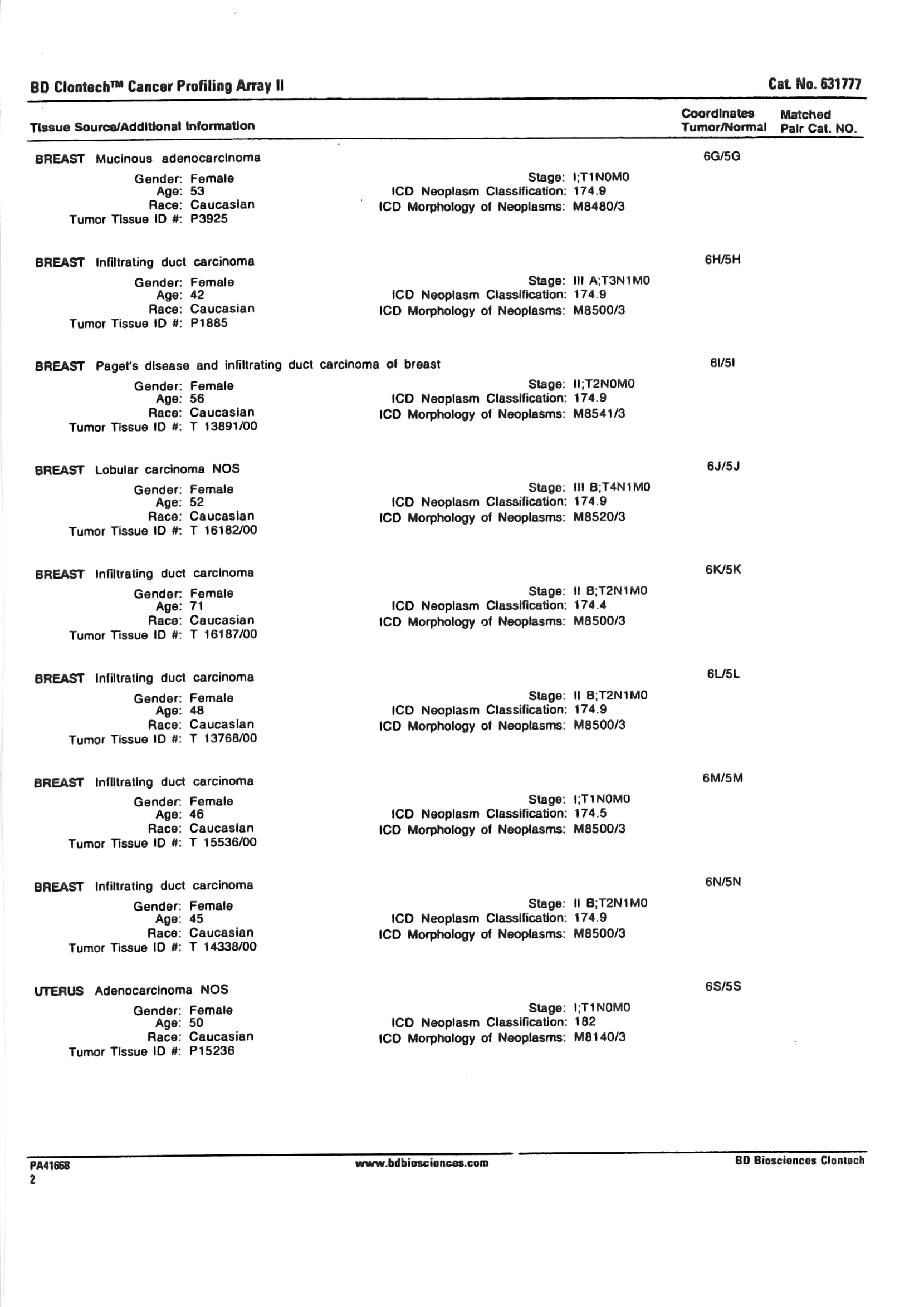


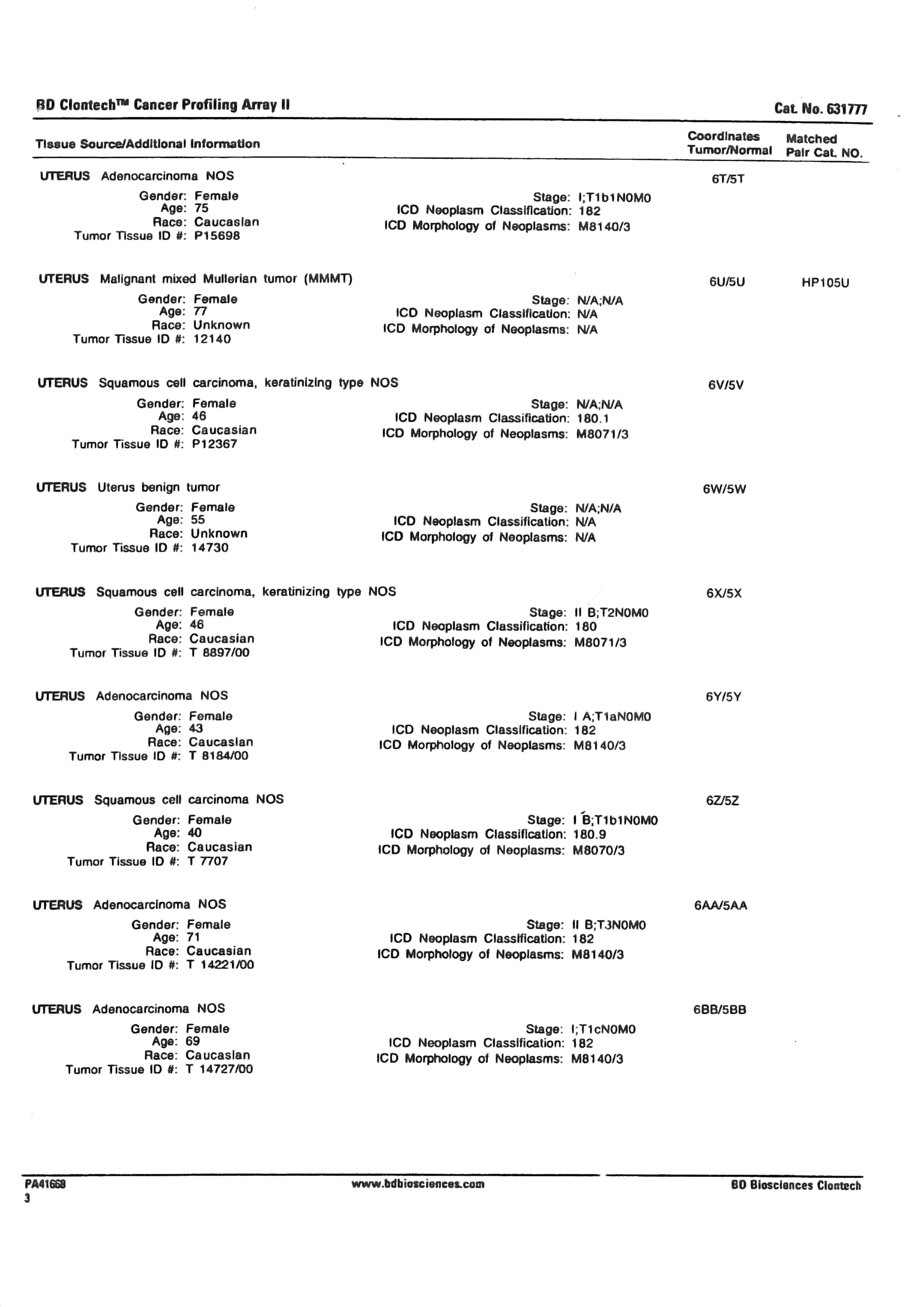


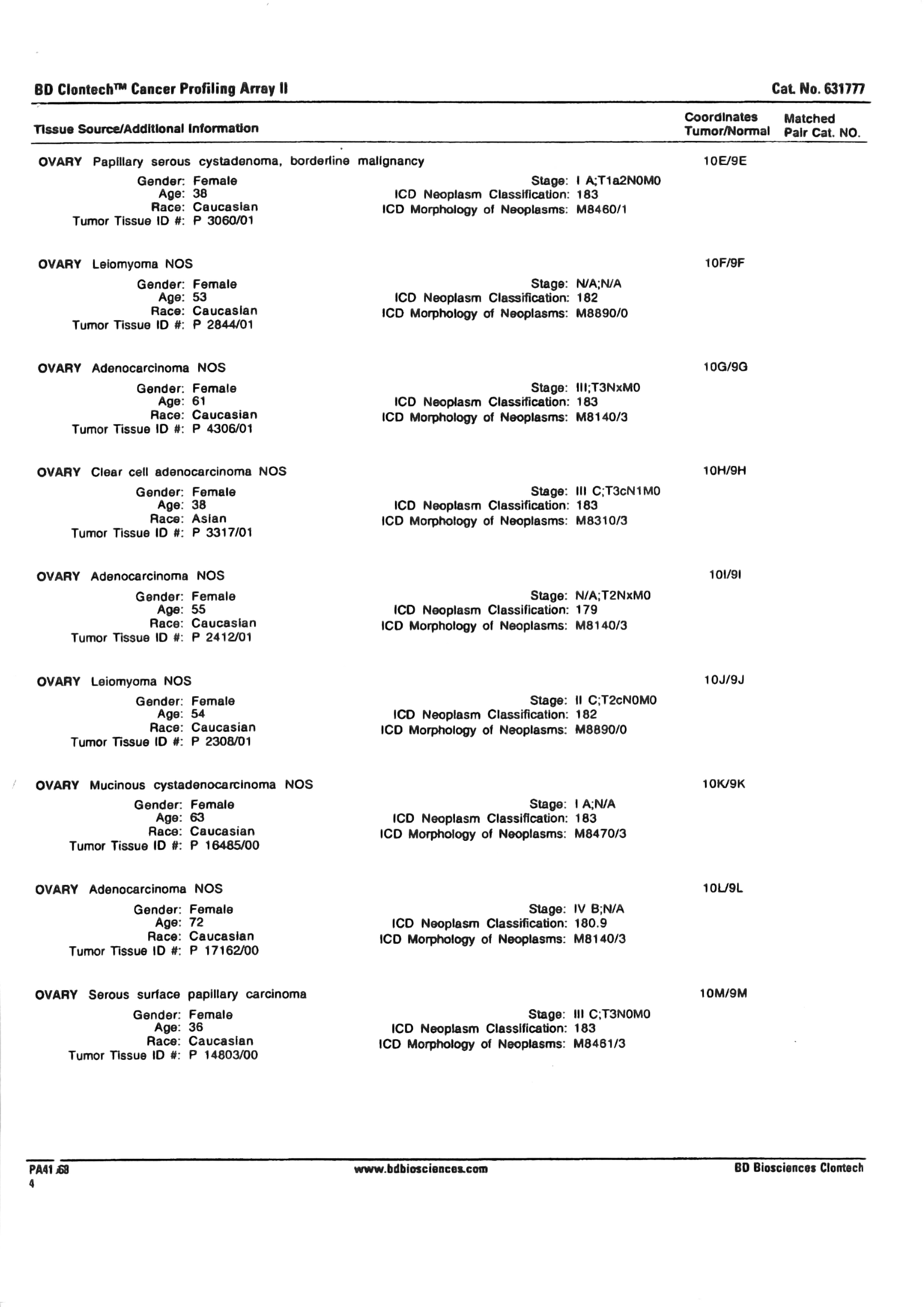


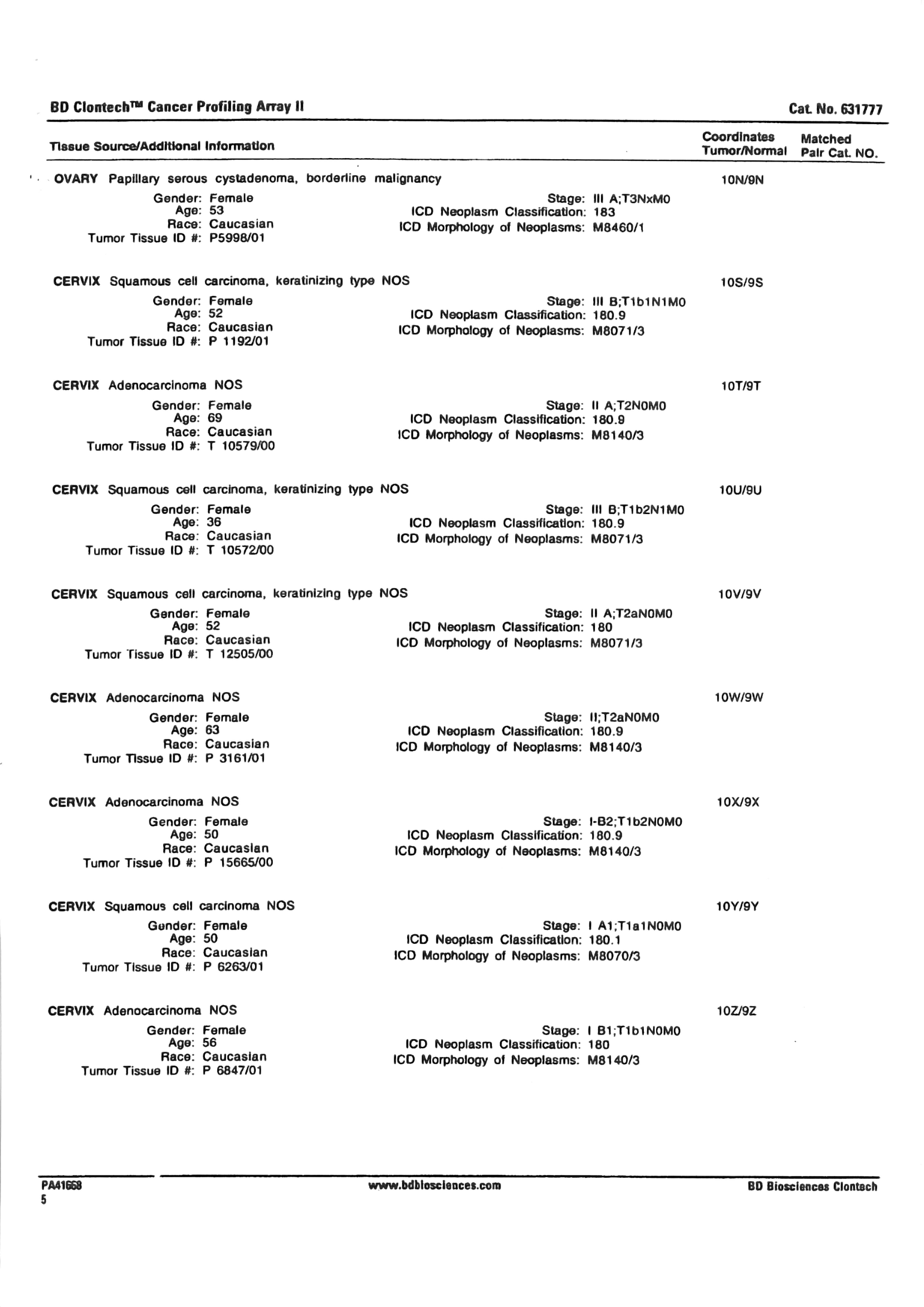


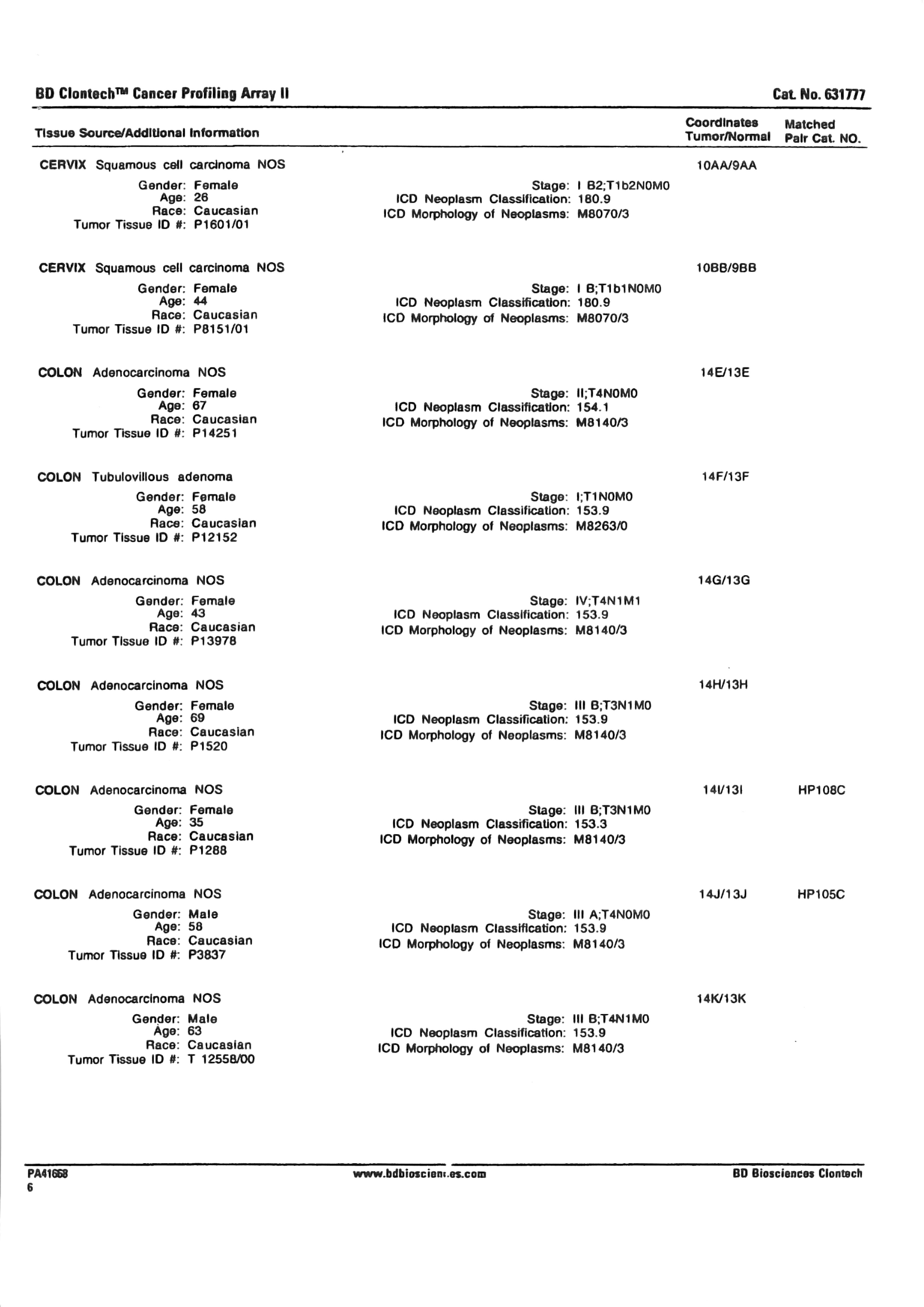


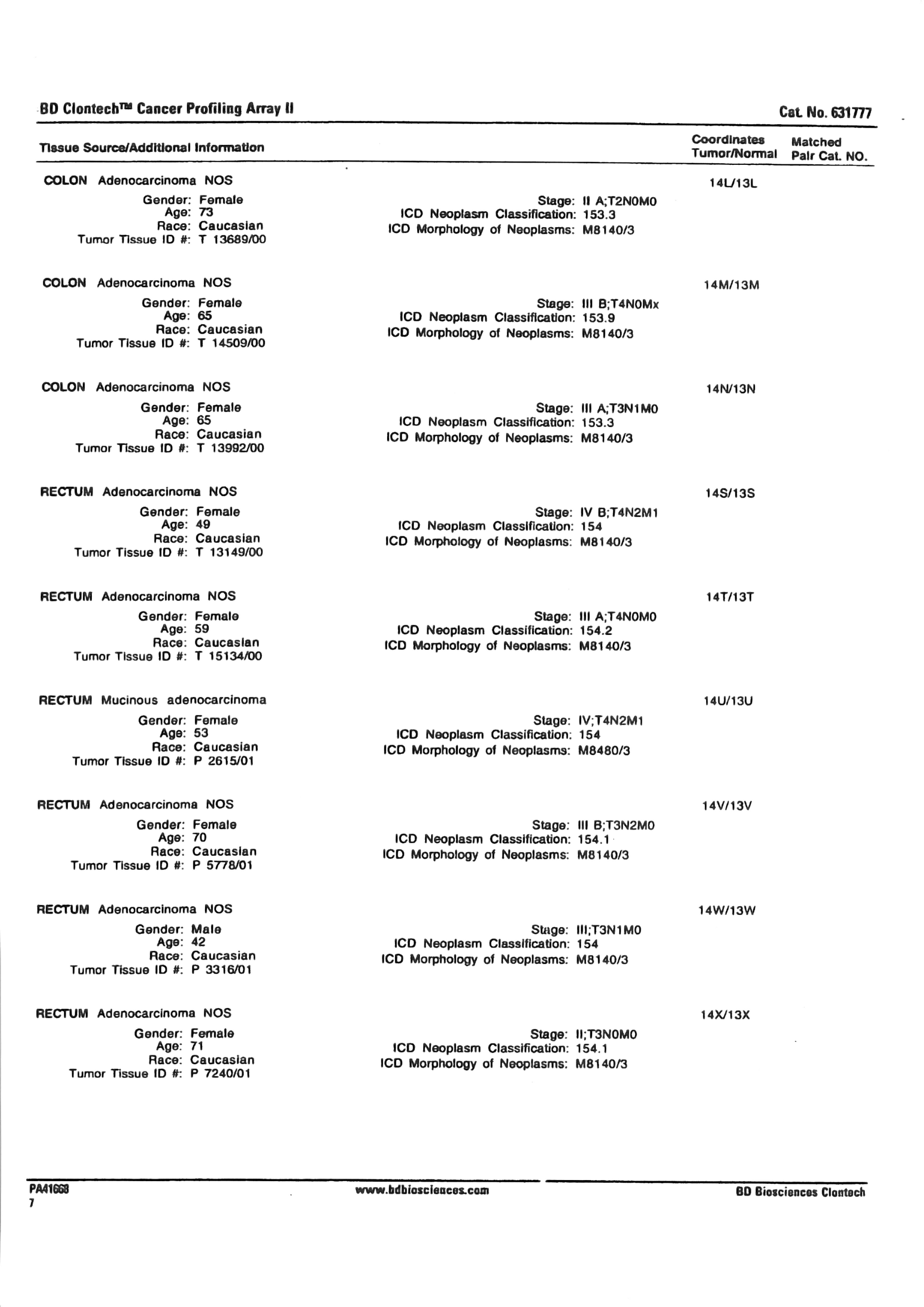


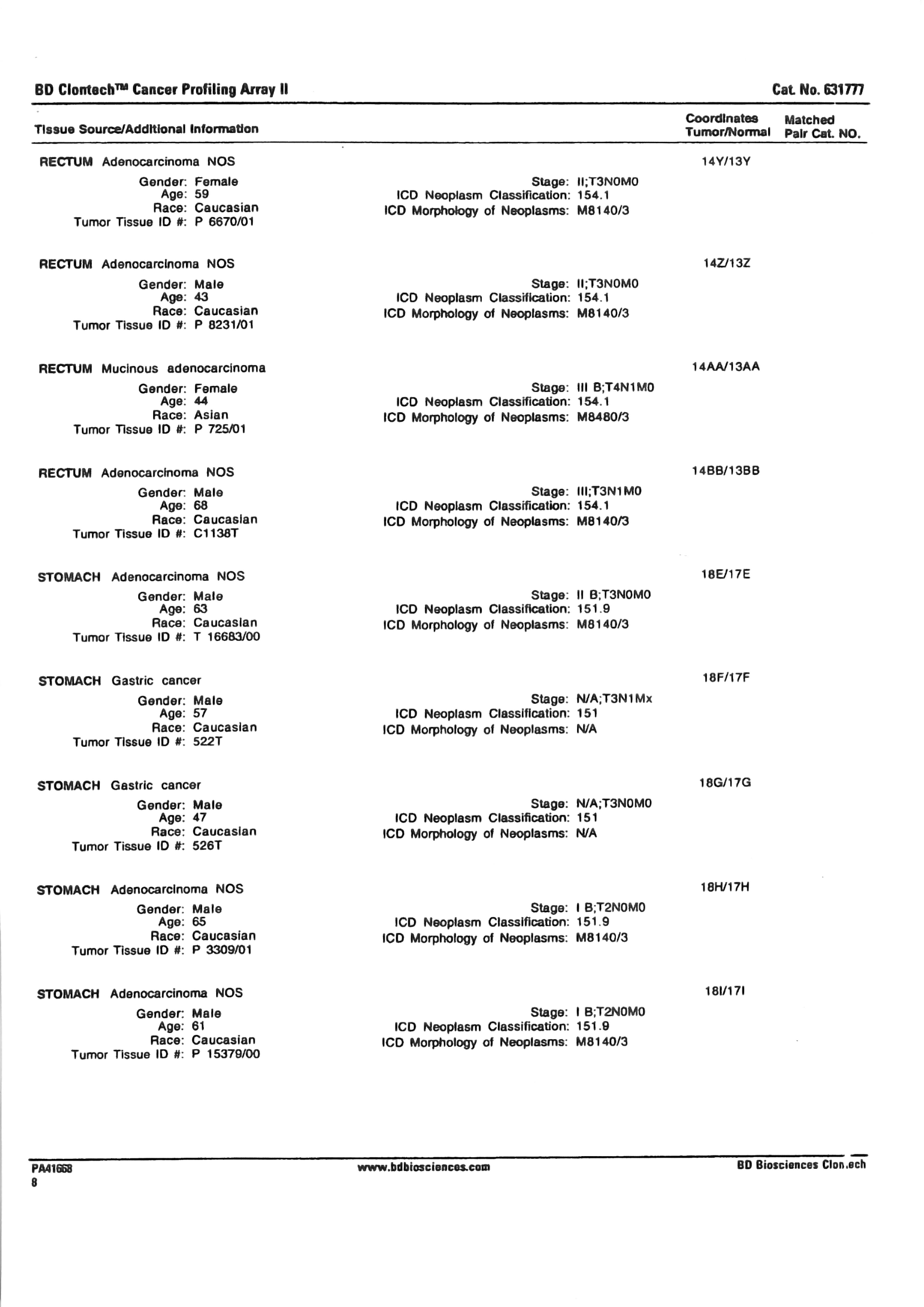


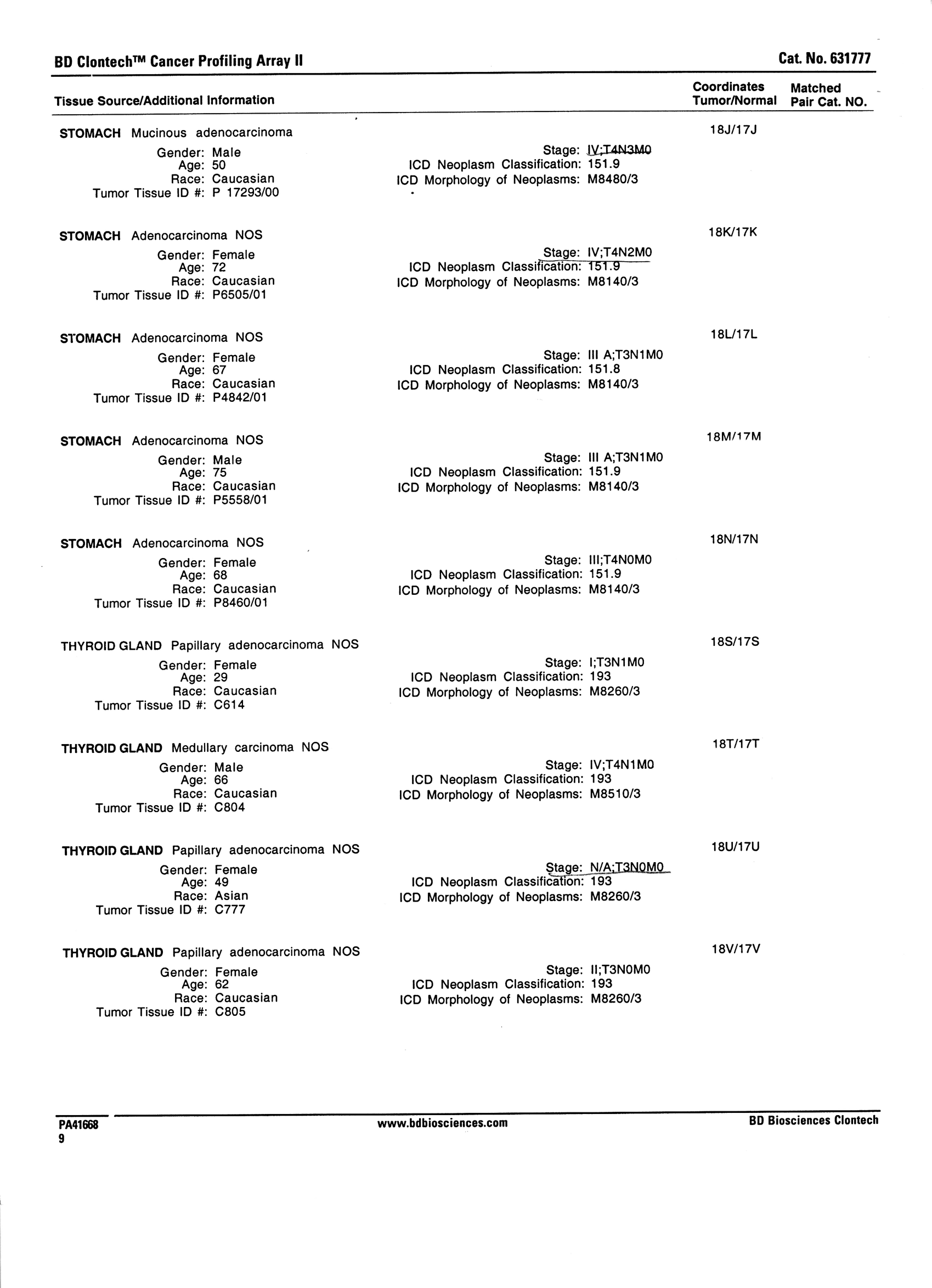


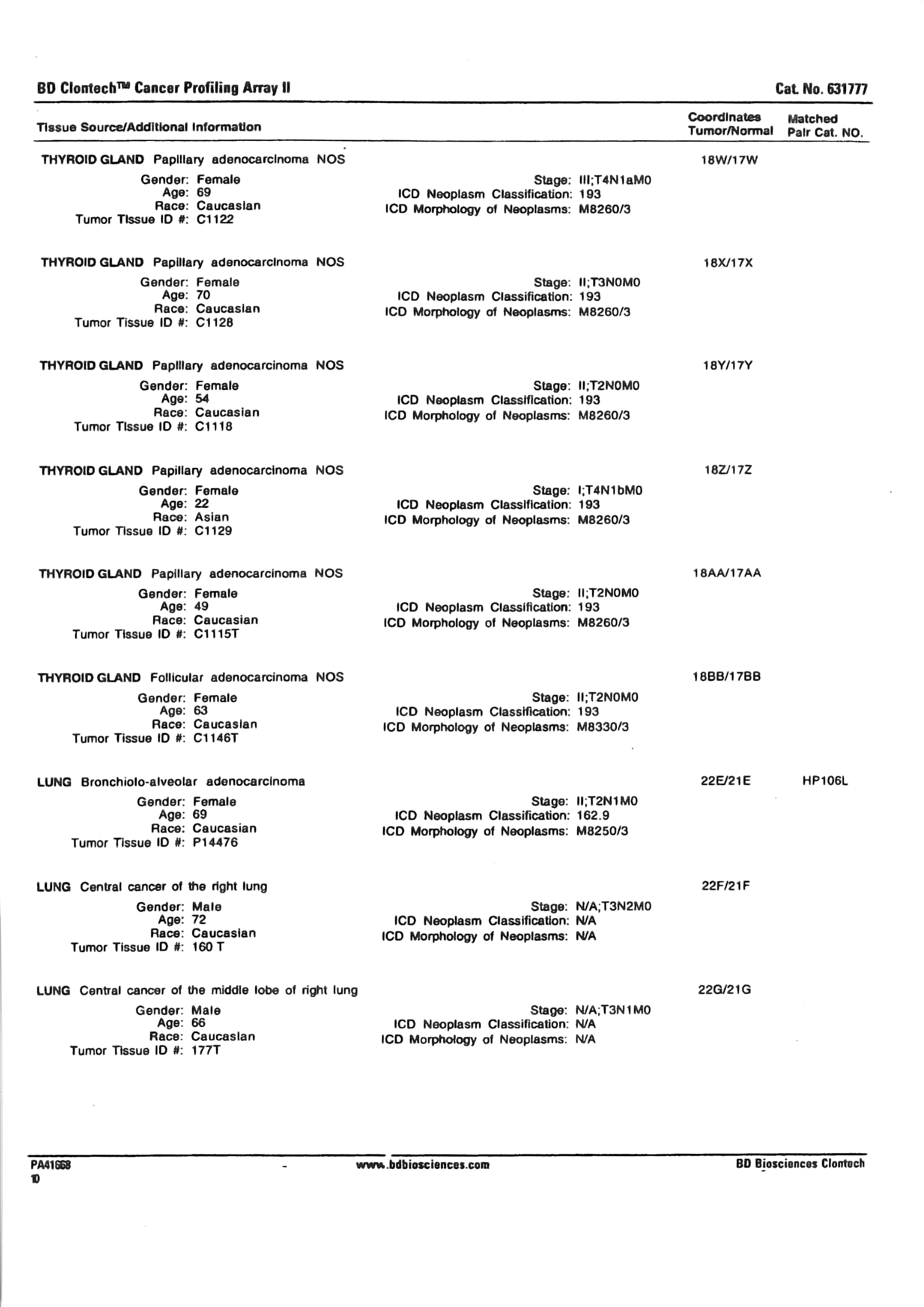


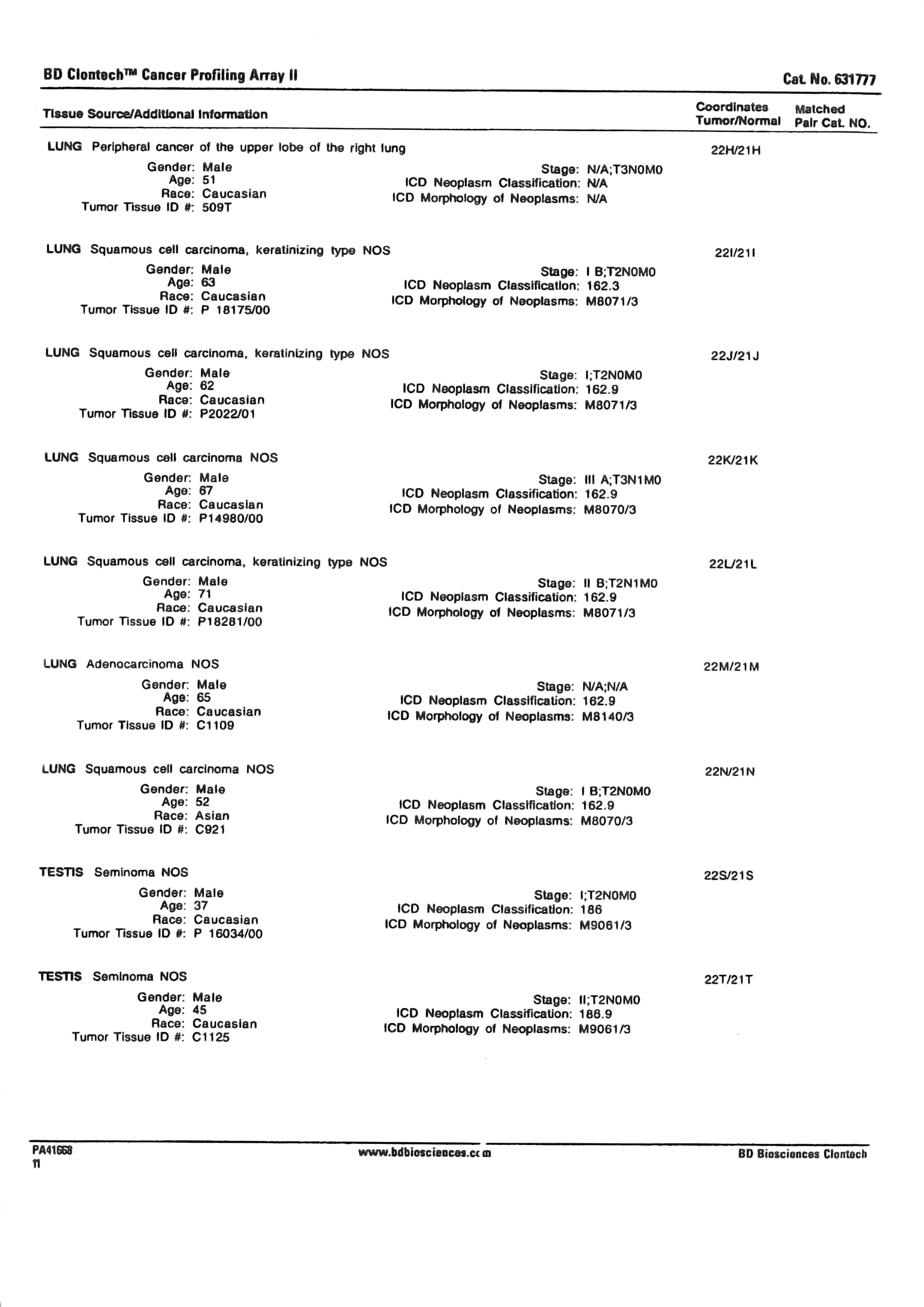


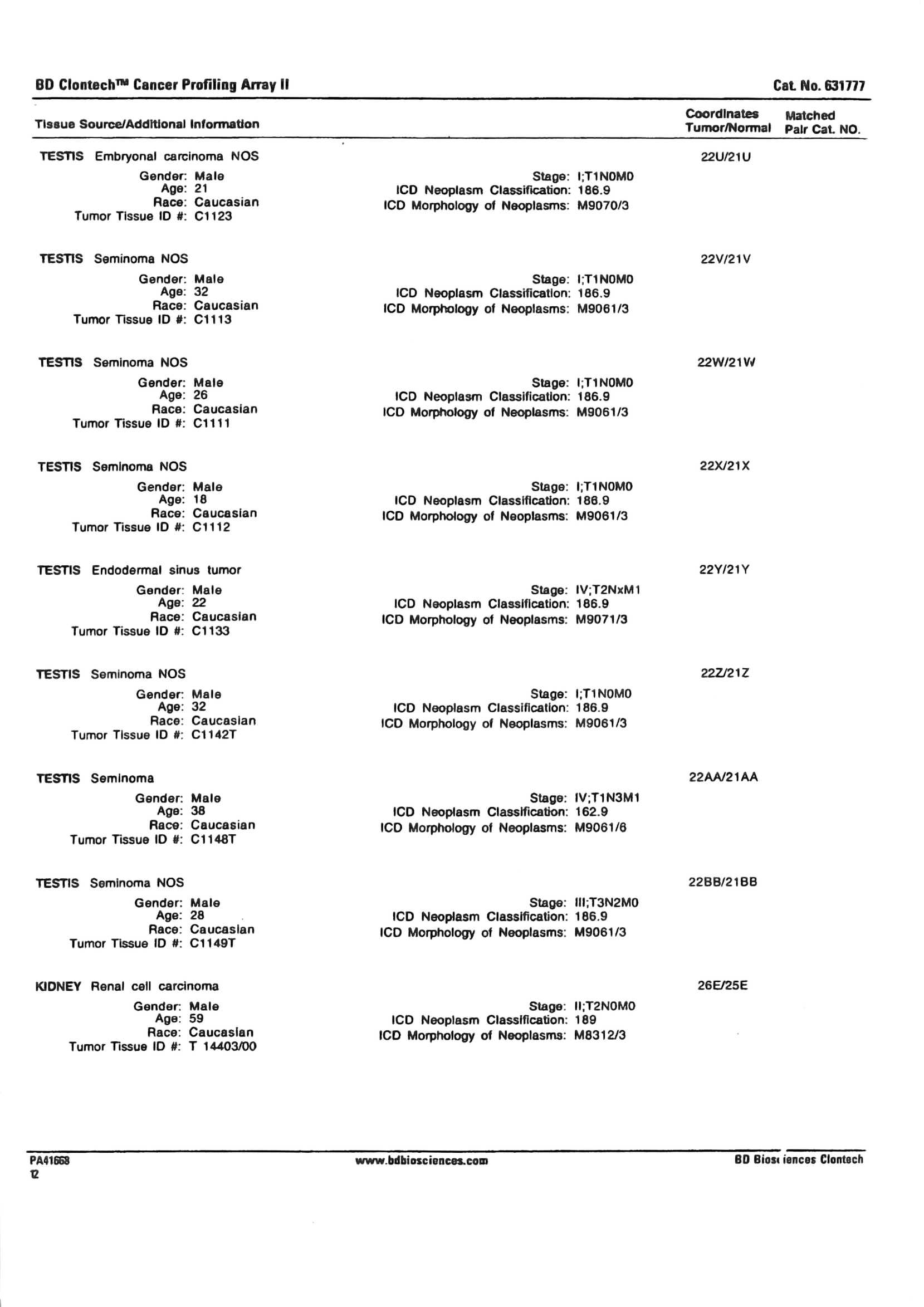


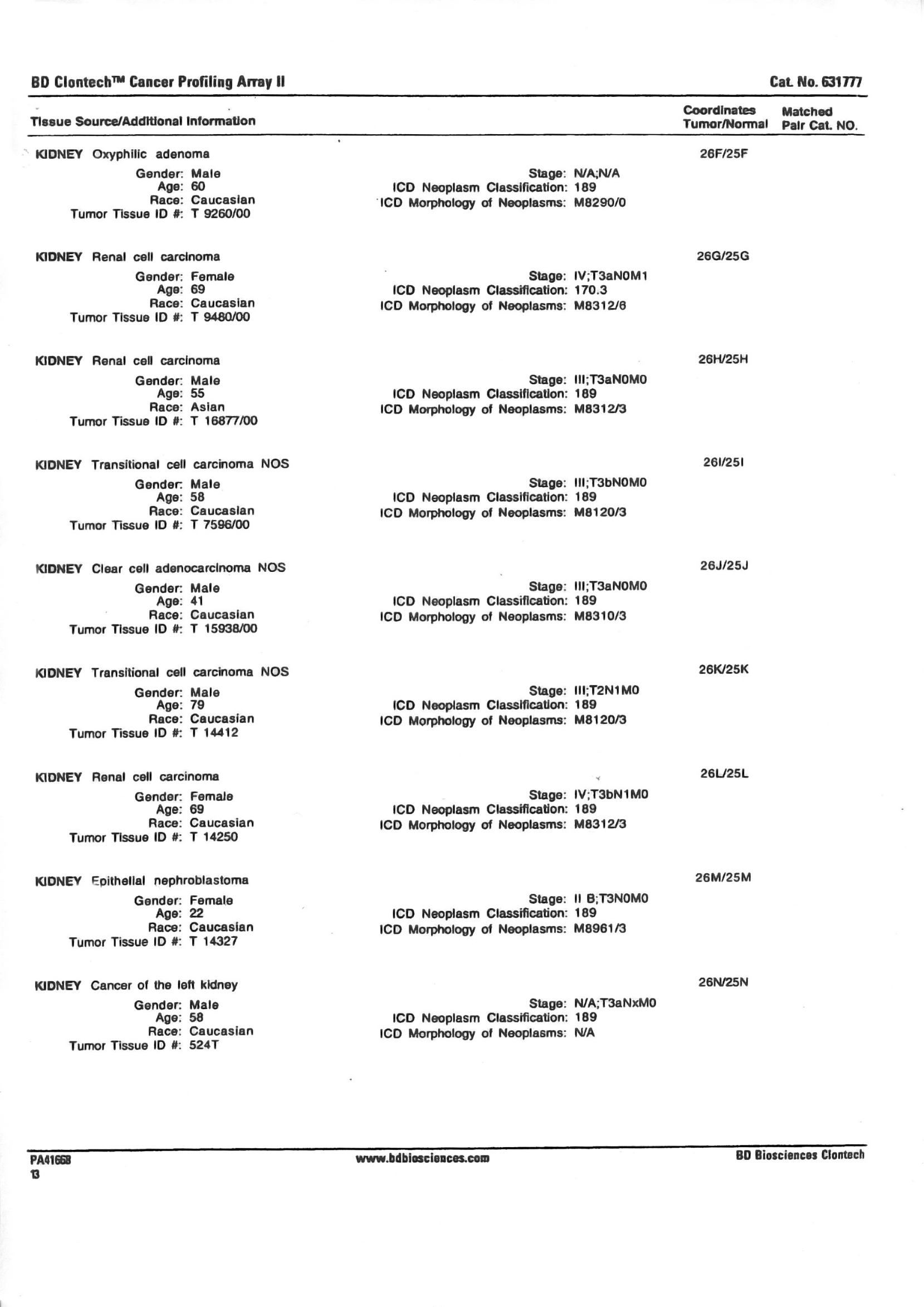


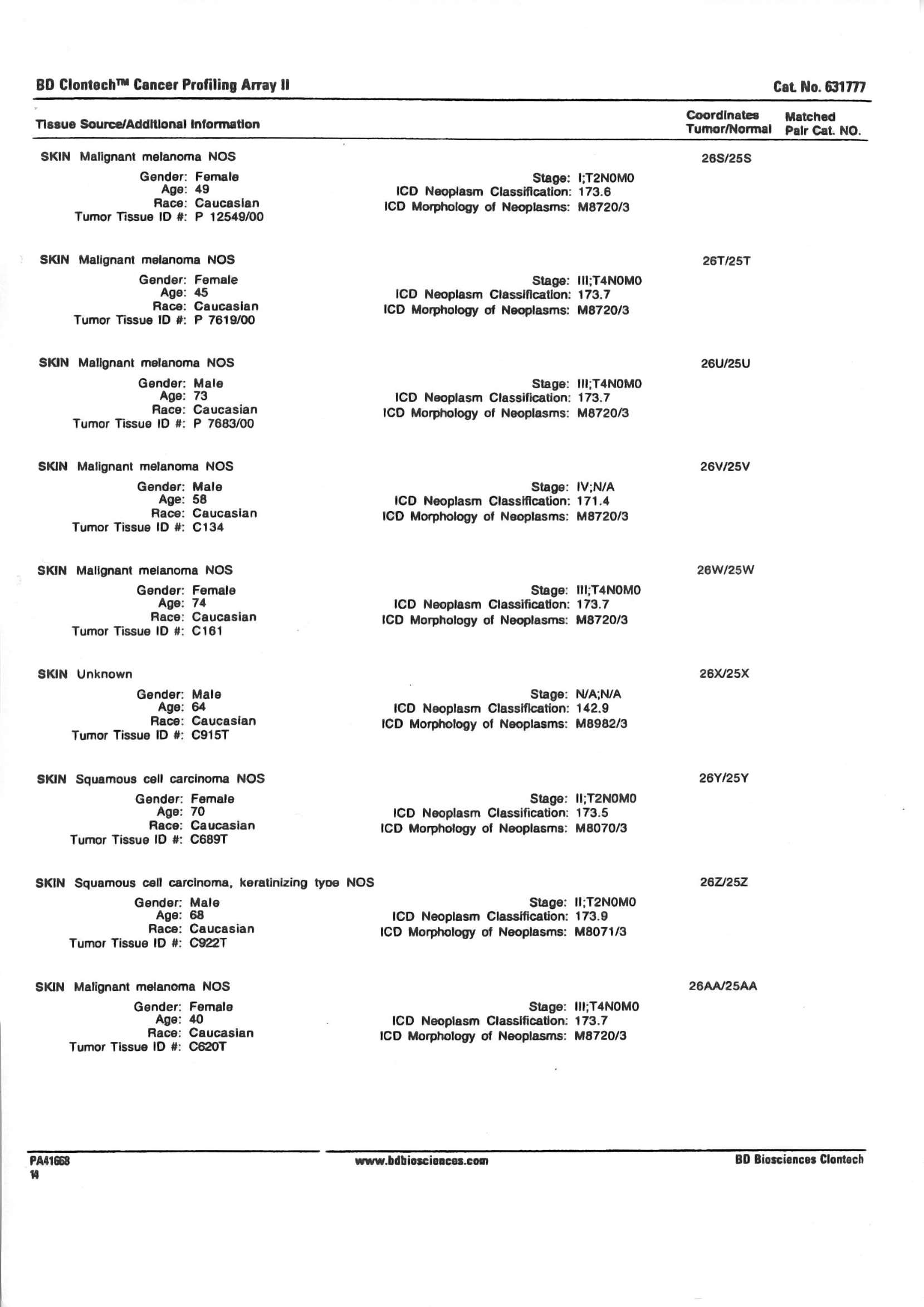


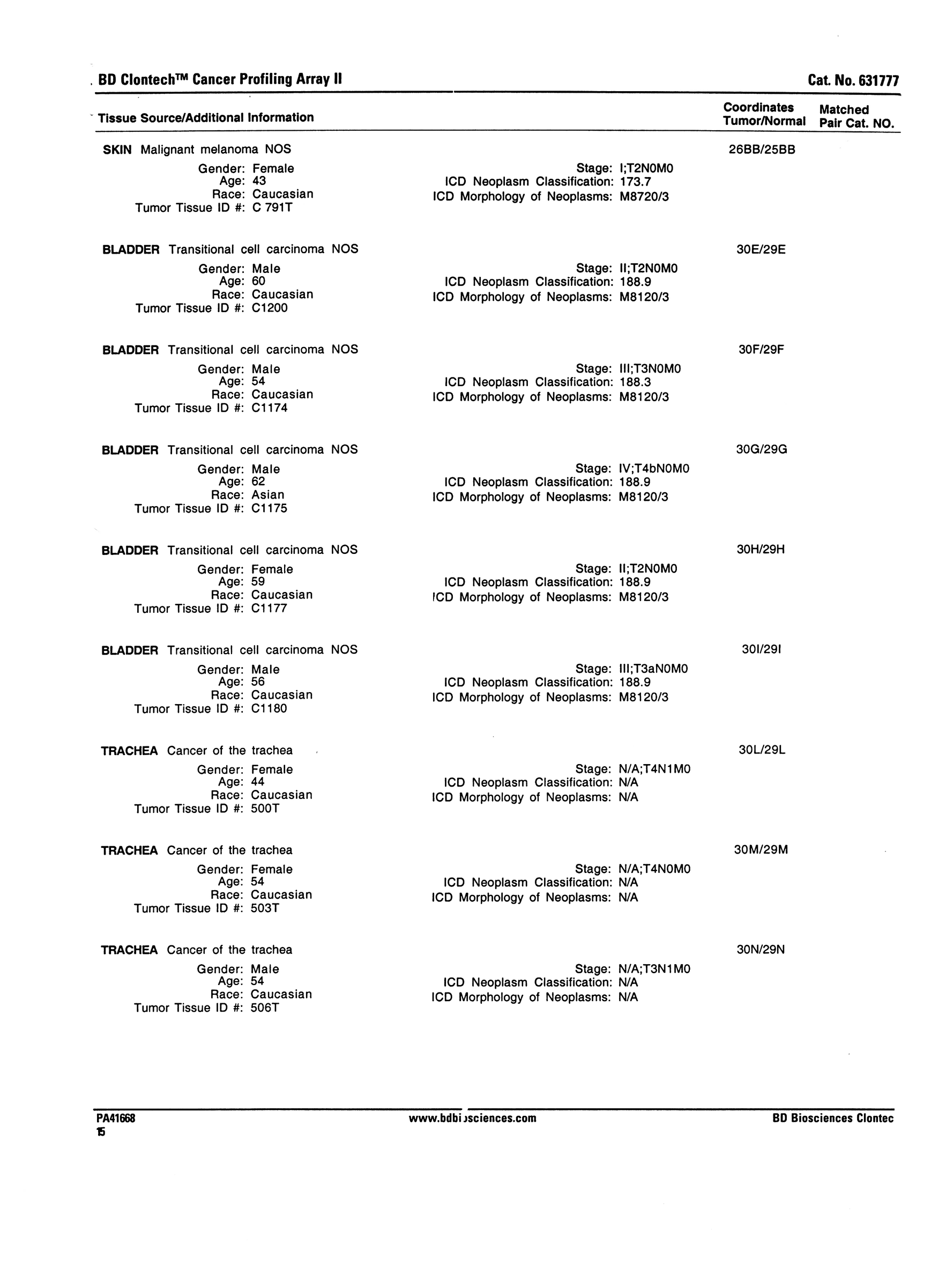


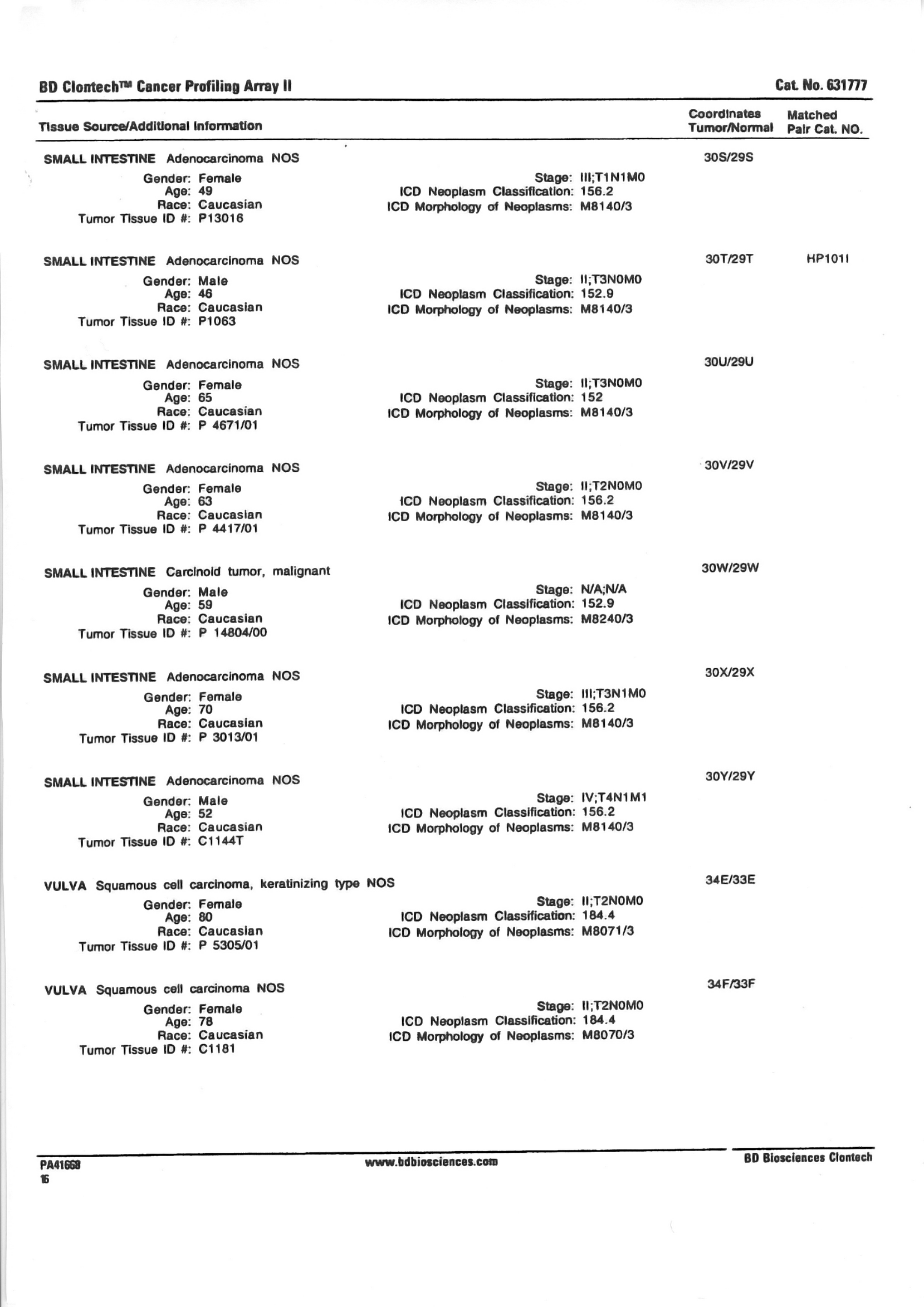


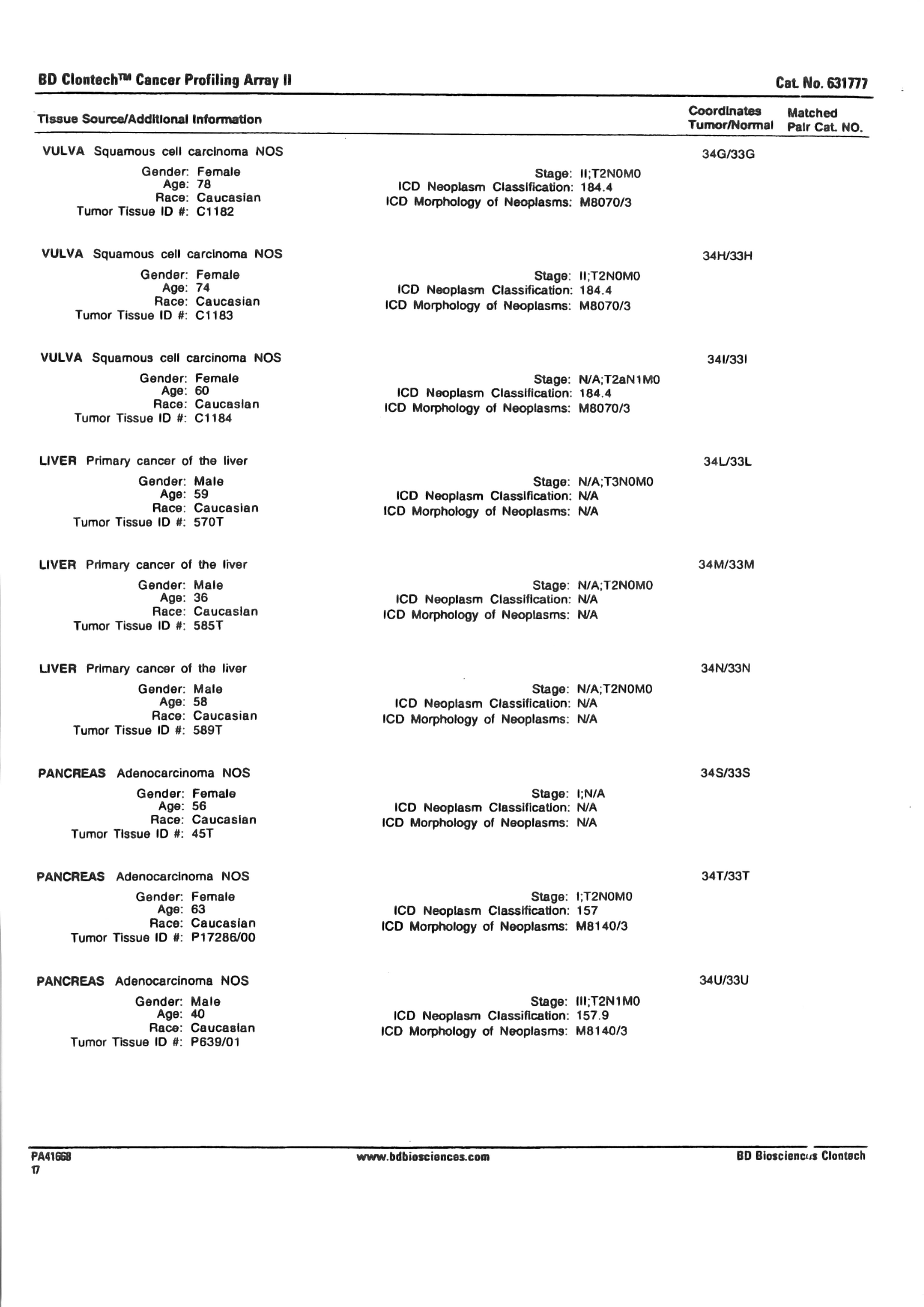


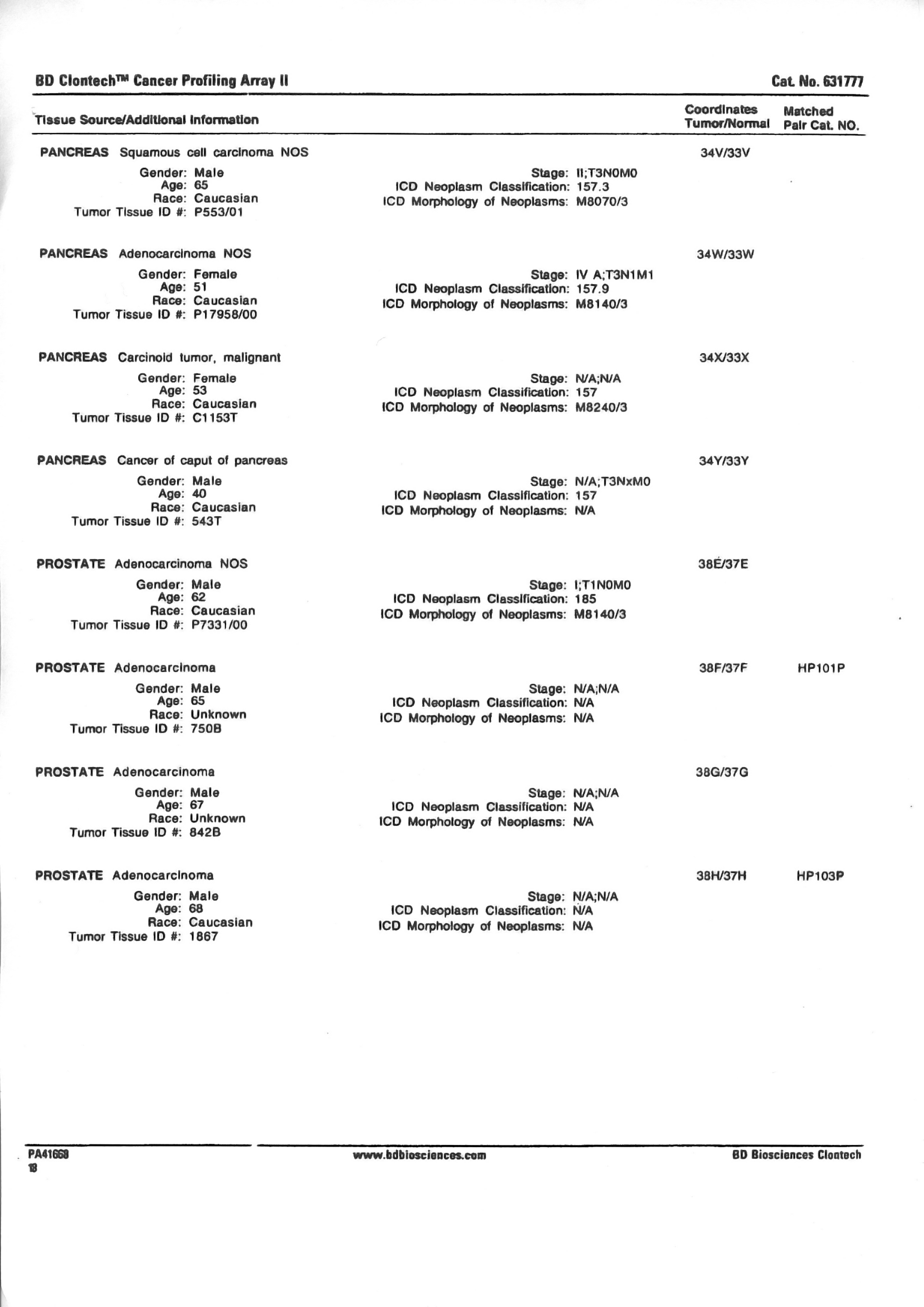


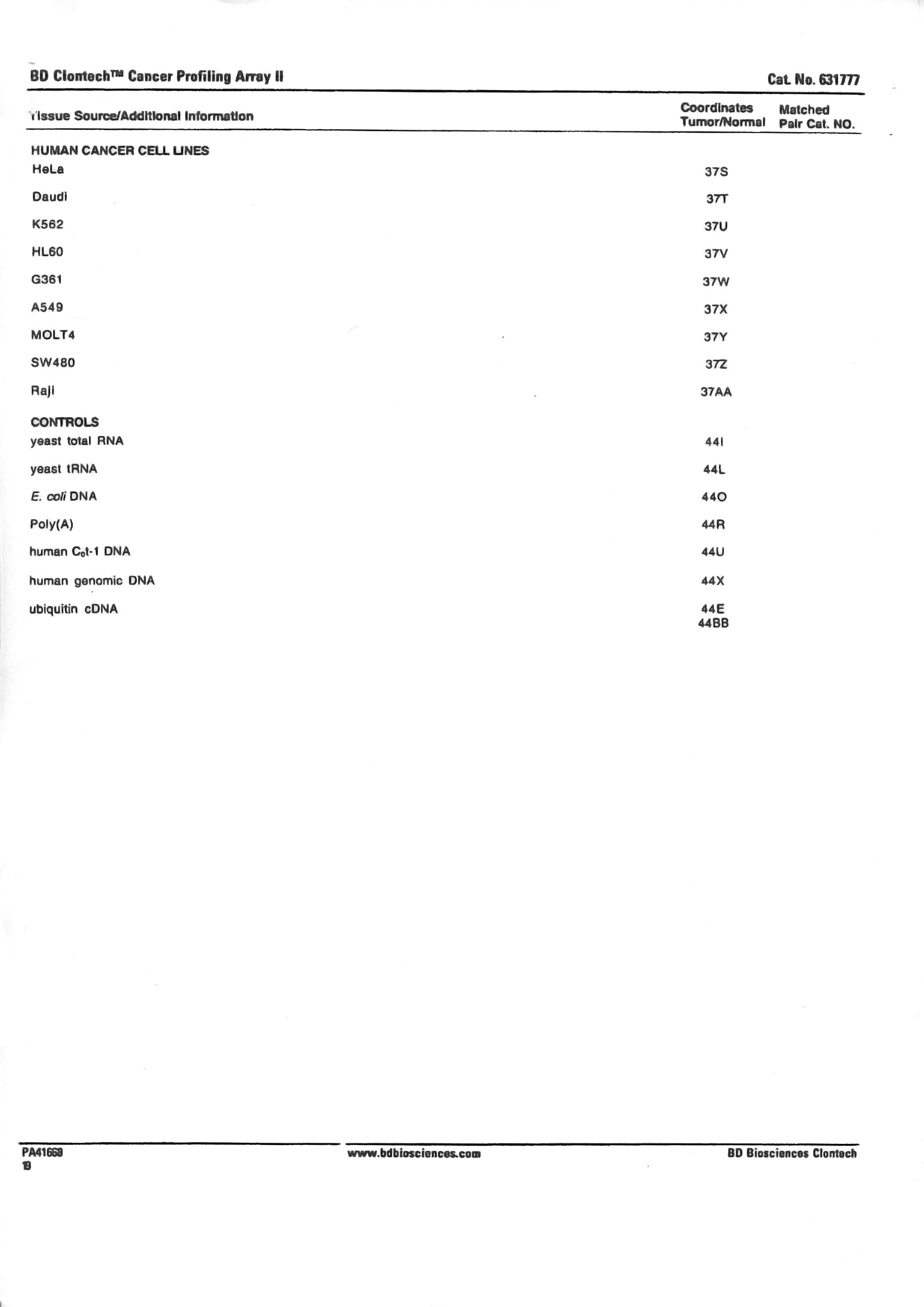

Supplement: Arrays Information S1 — This archive contains detailed information about localization and clinical characteristics of all the tumors (histological type, tumor size, stage, presence of metastases etc.). (DOC) [file pone.0015612.s001.doc]
